# Influence of historical changes in tropical reef habitat on the diversification of coral reef fishes

**DOI:** 10.1038/s41598-021-00049-4

**Published:** 2021-10-20

**Authors:** Fabien Leprieur, Loic Pellissier, David Mouillot, Théo Gaboriau

**Affiliations:** 1 UMR MARBEC (CNRS, IRD, IFREMER, UM), Université de Montpellier, Place Eugène Bataillon, 34095 Montpellier Cedex 5, France; 2 Institut Universitaire de France, Paris, France; 3 Landscape Ecology, Institute of Terrestrial Ecosystems, ETH Zürich, 8092 Zurich, Switzerland; 4 Swiss Federal Research Institute WSL, 8903 Birmensdorf, Switzerland; 5 Department of Computational Biology, University of Lausanne, 1015 Lausanne, Switzerland

**Keywords:** Phylogeny, Phylogenetics, Speciation, Biodiversity, Evolutionary ecology, Palaeoecology

## Abstract

Past environmental changes are expected to have profoundly impacted diversity dynamics through time. While some previous studies showed an association between past climate changes or tectonic events and important shifts in lineage diversification, it is only recently that past environmental changes have been explicitly integrated in diversification models to test their influence on diversification rates. Here, we used a global reconstruction of tropical reef habitat dynamics during the Cenozoic and phylogenetic diversification models to test the influence of (i) major geological events, (ii) reef habitat fragmentation and (iii) reef area on the diversification of 9 major clades of tropical reef fish (Acanthuridae, Balistoidea, Carangoidea, Chaetodontidae, Haemulinae, Holocentridae, Labridae, Pomacentridae and Sparidae). The diversification models revealed a weak association between paleo-habitat changes and diversification dynamics. Specifically, the fragmentation of tropical reef habitats over the Cenozoic was found to be a driver of tropical reef fish diversification for 2 clades. However, overall, our approach did not allow the identification of striking associations between diversification dynamics and paleo-habitat fragmentation in contrast with theoretical model’s predictions.

## Introduction

The history of earth is characterized by a succession of major environmental changes that profoundly shaped the diversity of life^1,2^. For instance, the breakup of the Pangaea and the successive phases of isolation and connection between continents during the Cretaceous (− 140 Mya) strongly influenced past biodiversity dynamics^3,4^ and also left a marked imprint on the current composition of species assemblages worldwide^4–6^. Similarly, major climate changes that occurred during the Cenozoic period (− 66 Mya), such as the rapid global warming at the Paleocene-Eocene boundary or the repeated periods of glaciation during the Quaternary period (− 2.6 Mya), also strongly affected species distribution^7,8^. A large number of studies suggested that these past changes strongly influenced the diversification of plants and animals, e.g. with a marked increase in clade species richness during warming periods^9,10^.

Early studies testing the influence of past environmental changes on diversification did not explicitly account for it when inferring diversification rates from phylogenies. Instead, studies employed a descriptive approach by identifying times where diversification changes and associate them temporally with major environmental changes based on simple visual inspections^11,12^. To tackle this issue, Condamine et al. proposed an environmental-dependence diversification model derived from time-dependent diversification models^13,14^. This model can be used to test whether speciation and extinction vary as a function of changes in a particular paleo-environmental variable that is time-dependent^15^. Using this approach, recent studies identified a strong influence of past-environmental changes on the estimation of speciation and extinction rates for several independent clades. For instance, Claramunt and Cracraft^3^ reported a negative relationship between both speciation and extinction rates in the modern avifauna and the global paleotemperature over the late Cretaceous and the Cenozoic, hence confirming the visual inspection of the relationship between the net diversification rate and the paleotemperature. Then, Condamine et al.^16^ found that high extinction rates in birdwing butterflies of the Indo-Australian Archipelago occurred during periods of elevated sea level and global warming. In a study of the diversification of Andean bellflowers, Lagomarsino et al.^17^ showed that speciation rates increased with Andean elevation, while extinction rates decreased during global cooling. More recently, Rolland and Condamine^18^ showed that palaeotemperatures have positively impacted the diversification of amphibians at the global scale. In addition, spatially explicit process-based models primarily driven by past environmental changes were found to predict observed patterns of biodiversity, including species richness and β-diversity gradients^4,19–22^.

In the marine realm, tropical coral reefs harbour an exceptional diversity of fishes, hosting one third of all marine fish species while only covering 0.1% of the ocean’s surface^23^. Numerous studies combined molecular phylogenetics and the fossil record to understand the origin and tempo of fish diversification in coral reefs^24–28^. Their results suggest that the development of a complex mosaic of reef habitats in the Indo-Australian Archipelago (IAA) during the Miocene, may have promoted cladogenesis, and provided a refuge from extinction. Indeed, in the Early Miocene (~ 23–16 mya), the Australian plate made contact with the submerged Sundaland and this collision led to closure of the shallow reefs between Sundaland and Australia^29^, which increased reef area and the complexity of coral reefs throughout the region. More recently, Pellissier et al.^8^ and Gaboriau et al.^30^ reported that the Quaternary glaciations (− 2.6 mya) may have played a major role in the diversification of coral reef fishes. During cycles of global cooling, coral reef habitats were only maintained in some refugia, where environmental conditions remained favourable for their development, but their isolation from each other have probably promoted allopatric or parapatric speciation^31,32^. The loss of coral reef habitats during the Quaternary likely induced numerous extinctions in coral reef fish lineages but fossil evidences are currently lacking. Last, older geological events may have likely contributed to the diversification of coral reef fishes. Indeed, recent studies suggested that plate tectonics induced the fragmentation of shallow tropical reefs of the Tethys (i.e. the largest tropical paleo-ocean) during the Late Cretaceous (~ 100–65 Ma) and the Paleogene (~ 65–23 Ma), which promoted multiple vicariance events in the tropical marine fauna^4,33^. Despite the knowledge gained from these studies, a clear and quantitative assessment of how earth history events have influenced the diversification of coral reef fishes is still lacking.

**Figure 1:**
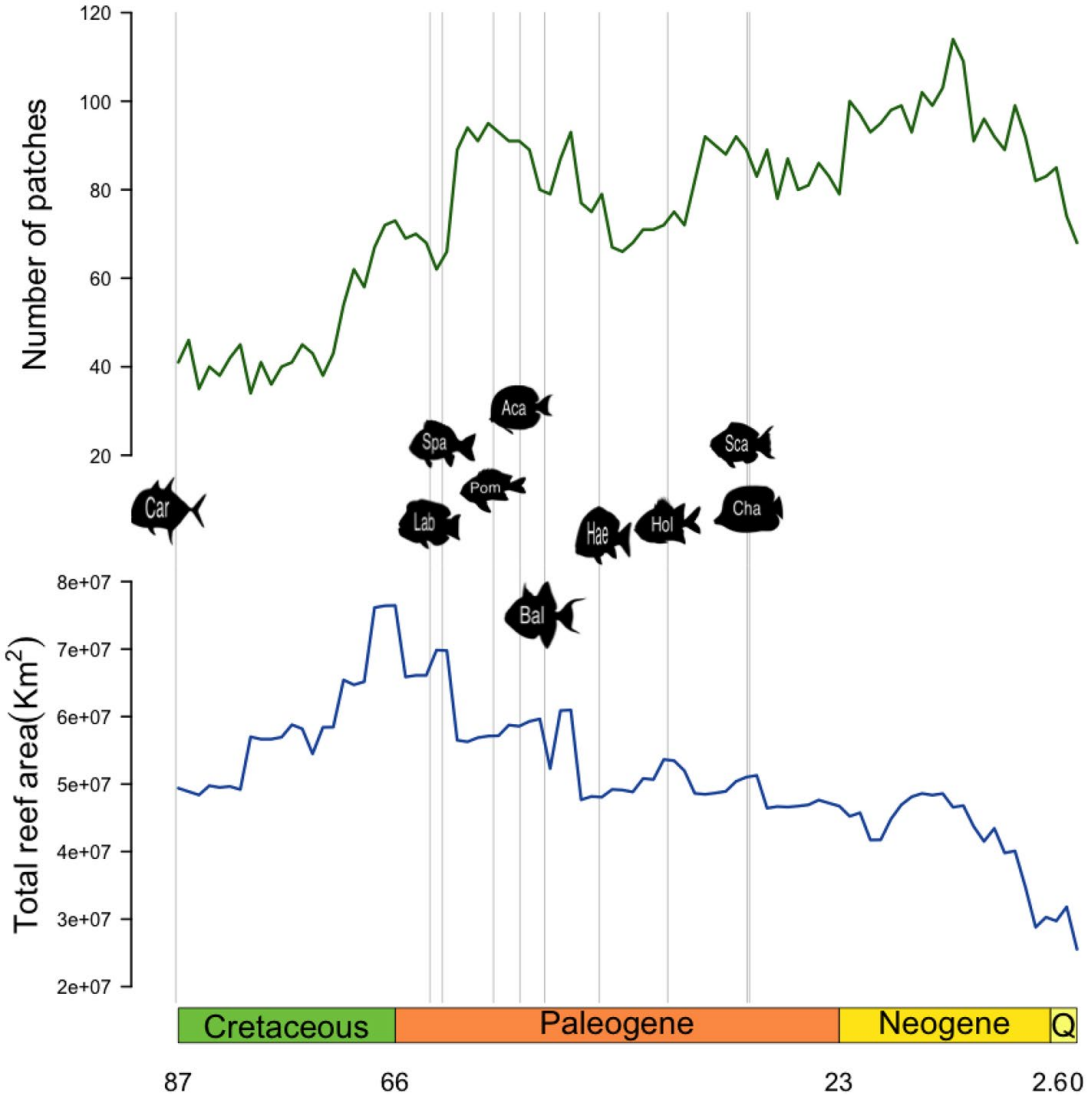
Variations in number of coral reef patches and total coral reef area through time. The grey lines correspond to the estimated crown age of the phylogenies included in the analyses. *Aca* Acanthuridae, *Bal* Balistoidea, *Car* Carangoidea, *Cha* Chaetodontidae, *Hae* Haemulinae, *Hol* Holocentridae, *Lab* Labridae, *Pom* Pomacentridae, *Spa* Sparidae.

In the present study, we first tested whether major earth history events such as the Quaternary glaciations and the collision of the Australian plate with the submerged Sundaland in the Early Miocene impacted reef-fish diversification to the extent that their signal could be identified from phylogenies, using a *birth–death-shift* model of evolution (TreePar^34^). We then assessed whether the fragmentation of shallow tropical reef habitats (fragmentation hypothesis) and their change in surface area (area hypothesis) through the past 100 Ma (Fig. 1) influenced the diversification of coral reef fishes. To do so, we applied the approach proposed by Condamine et al.^15^ by fitting a series of environmental birth–death models that we compared to a simple birth–death model. We focused on 9 clades of coral reef fishes for which recent time-calibrated phylogenetic trees have been published: Acanthuridae, Balistoidea, Carangoidea, Chaetodontidae, Haemulinae, Holocentridae, Labridae, Pomacentridae, Sparidae. These clades are representative of the global coral reef fish fauna as they are speciose and abundant on tropical coral reefs and display a large variety of functions^35^.

## Results

### Timing of diversification

Most taxa investigated in this analysis originated during the Paleogene, a period with high amplitude variation of fragmentation. Based on consensus trees, only Carangoidea and Chaetodontidae were estimated to have no shift of diversification rate during their lifespan (Table 1). The other taxa were estimated to have one shift of diversification (Table 1) or two (Holocentridae). There was low congruence between consensus trees and trees taken from the posterior distributions for all families. However, constant diversification models were rejected in more than 95% posterior trees of Acanthuridae, Haemulinae, Holocentridae and Labridae (Table 1).

**Table 1:**
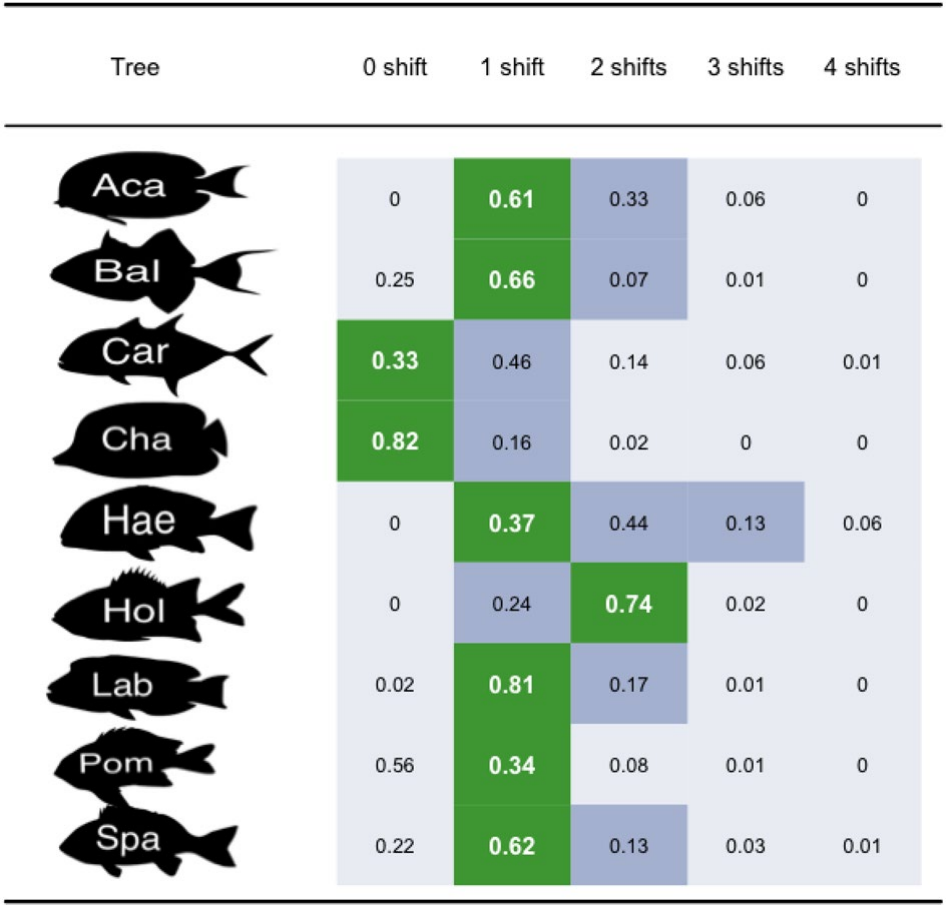
Models selected by the TreePar analysis.

For each phylogeny, the cell in green represents the optimal number of shifts estimated on the consensus tree; The cells in dark grey represent the models based on the consensus tree that have a $\Delta AICc$ lower than two and the cells in light grey represent the models based on the consensus tree, that are rejected by the AICc approach. The proportions represent the frequency of selection of each model in the posterior distributions with the AICc approach. *Aca* Acanthuridae, *Bal* Balistoidea, *Car* Carangoidea, *Cha* Chaetodontidae, *Hae* Haemulinae, *Hol* Holocentridae, *Lab* Labridae, *Pom* Pomacentridae, *Spa* Sparidae.

**Figure 2:**
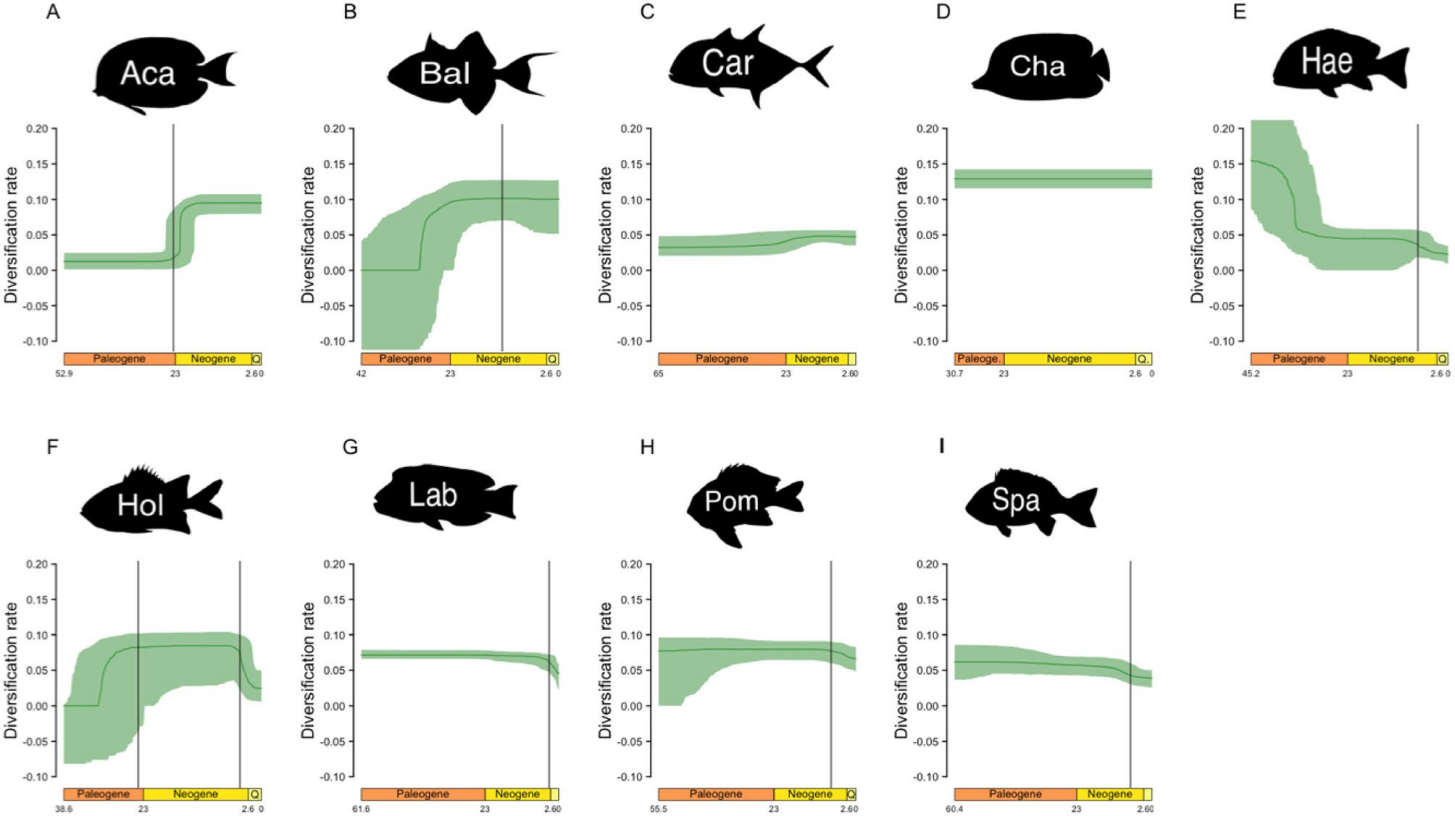
Diversification rates through time estimated under the *birth–death-shift* model. The dark green line represents the median diversification rate of the trees from the posterior distribution calculated every 100,000 years. The light green area represents the first and the ninth decile of the diversification rate distribution estimated every 100,000 years. We present the estimated diversification rates based on the posterior distribution of trees to represent the errors linked to phylogenetic and dating uncertainties. Thus, clades that are expected to display a constant diversification rate based on their consensus tree, can display temporal variation in diversification rates here. The dark grey line represents the date of the diversification rate shift estimated on consensus trees. *Aca* Acanthuridae, *Bal* Balistoidea, *Car* Carangoidea, *Cha* Chaetodontidae, *Hae* Haemulinae, *Hol* Holocentridae, *Lab* Labridae, *Pom* Pomacentridae, *Spa* Sparidae.

Two remarkable shifts of diversification were repeatedly identified in both posterior distributions and consensus trees. The first remarkable shift is a burst of diversification between the late Paleogene and the early Neogene. This burst has been identified in the best fitting models of Acanthuridae and Holocentridae (see supplementary online information). Furthermore, the mean diversification rate of posterior distributions also suggested the presence of that shift in Acanthuridae, Balistoidea, Carangoidea and Holocentridae (Fig. 2). The second remarkable shift is a drop of diversification at the beginning of the Quaternary. It has been identified in the best fitting models of Haemulinae, Labridae, Pomacentridae and Sparidae (Table S1). It was also suggested by the mean diversification rate of trees from posterior distributions in Haemulinae, Holocentridae, Labridae and Sparidae (Fig. 2).

### Influence of past habitat changes on diversification

#### Fragmentation hypothesis

Within the lifespan of our taxa, we observe important variations in the number of patches representing habitat fragmentation with a peak in the late Neogene (Fig. 1). When fitted on consensus trees of Acanthuridae, Balistoidea and Holocentridae, the *environmental birth–death* model provides a better support than a pure *birth–death* model ($\Delta AICc>2,$ Table 2). Based on consensus trees of the others clades, goodness-of-fit of the pure *birth–death* model appears to be better than the *environmental birth–death model.* The results based on the posterior distribution of trees were not consistent with those based on consensus trees (Table 2). The pure *birth–death* model was rejected in 95% of the posterior trees in Acanthuridae, Haemulinae and Holocentridae and it was accepted in 95% of the posterior trees only for Pomacentridae (Table 2).

**Table 2:**
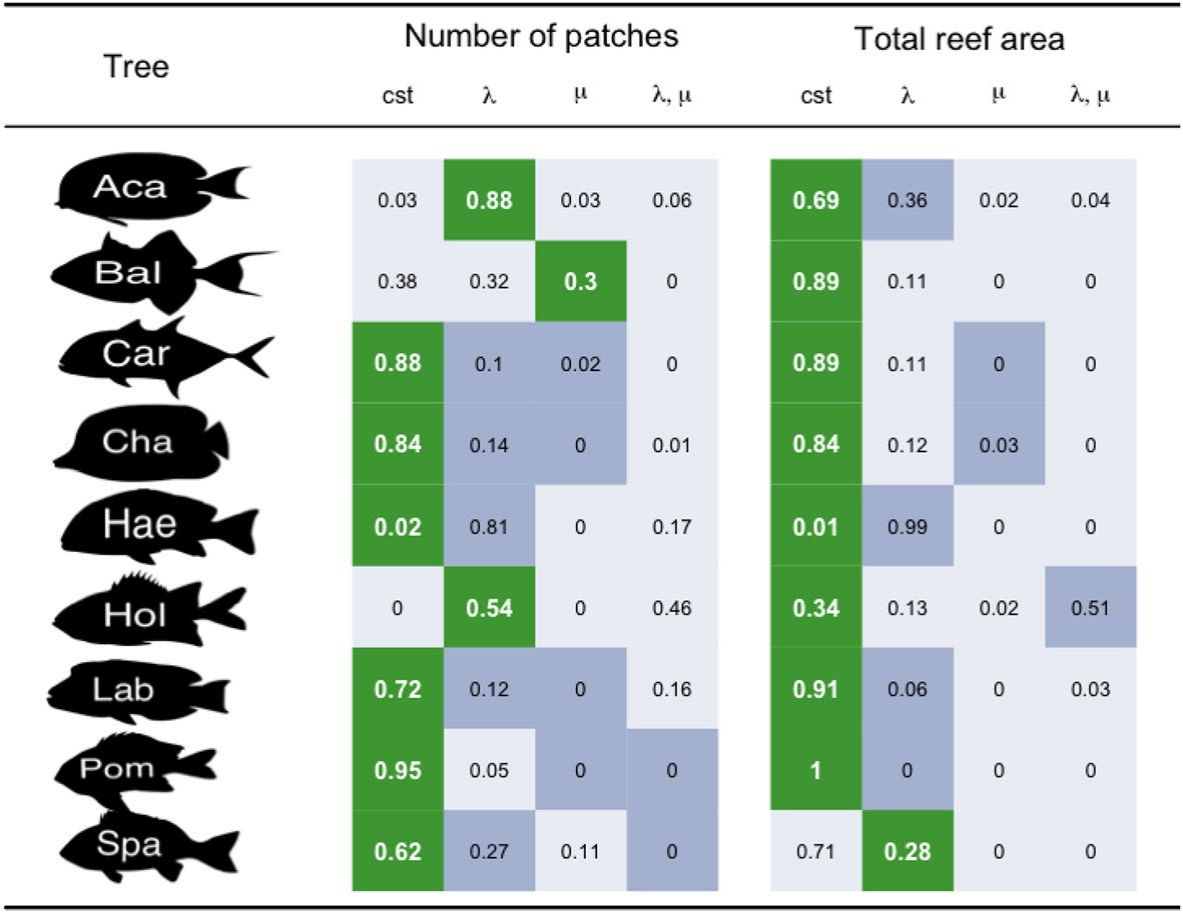
Influence of the paleo-environment on the speciation and extinction rates of each phylogeny.

The table is divided in two parts: one for the influence of the number of patch through time and one for the influence of the total are through. For each taxa, the cell in green represents the best model selected with the AICc approach on the consensus tree; The cells in dark grey represent the models based on the consensus tree, that have a $\Delta AICc$ lower than two; The cells in light grey represent the models based on the consensus tree, that are rejected by the AICc approach. The proportions represent the frequency of selection of each model in the posterior distributions with the AICc approach. For each environmental variable, the column ‘cst’ represents the model 1 (pure *birth–death*), the column ‘λ’ represents the models 2.1 and 2.2 (*environmental birth–death* with the speciation rate dependant on the environmental variable), the column ‘μ’ represents the models 3.1 and 3.2 (*environmental birth–death* with the extinction rate dependant on the environmental variable) and the column ‘λ,μ’ represents the models 4.1 and 4.2 (*environmental birth–death* with both speciation and extinction rate dependant on the environmental variable). *Aca* Acanthuridae, *Bal* Balistoidea, *Car* Carangoidea, *Cha* Chaetodontidae, *Hae* Haemulinae, *Hol* Holocentridae, *Lab* Labridae, *Pom* Pomacentridae, *Spa* Sparidae.

#### Area hypothesis

The total reef area peaks at the beginning of the Paleogene and drops several times until present day. We identified no influence of this variable on coral reef fish diversification. The pure *birth–death* model is better than the area-dependant model to explain all consensus trees, excepting Sparidae (Table 2). Overall, in 75.6% of the investigated trees, the pure *birth–death* model is a better model than the total reef area dependant model.

Results based on the posterior distributions are consistent with those based on the consensus trees except for Sparidae, for which the pure *birth–death* model is the most frequently selected model, and for Haemulinae, for which the *environmental birth–death* model is the most frequently selected model (Table 2).

## Discussion

Due to the weak support given to those hypotheses and the inconsistencies revealed by tree reconstruction’s posterior distributions, our study remains inconclusive about the role of plate tectonics over the Cenozoic on the diversification tropical reef fishes, even if varying responses were observed between them.

### Timing of diversification

Using the approach proposed by Stadler^34^ for studying the temporal dynamic of diversification, we identified two major diversification shifts for several tropical reef fish clades. The estimated dates of these shifts are clearly related to two major earth history events (Holocentridae, Balistoidea, Haemulinae : Oligocene; Acanthuridae, Carangoidea : early Miocene; Haemulinae, Holocentridae, Labridae, Pomacentridae, Sparidae: Quaternary), which were previously hypothesized to have played a major role in shaping the diversification of tropical reef fishes^4,8,25,36–38^.

The oldest shift, observed in the diversification rates of Acanthuridae, Balistoidea, Carangoidea, Haemulinae and Holocentridae (Fig. 2), correspond to the period that is marked by a succession of geological events that shaped the Indo-Australian Archipelago^33,37^. First, the transition between the Oligocene and the Miocene is marked by the progressive closure of the Tethys Ocean, and by the stepping-stone colonisation of the Indo-Pacific by Tethyan species^4,27^. Second, the collision between Australia and Southeast Asia at 23 Mya^37,39^ fostered the formation of many isolated archipelagos and shallow seas that started to host coral reefs^40^. These events have probably accelerated the diversification of reef-fish according to two main mechanisms: allopatric speciation between the Indo-Pacific and the Tethys Ocean^27,33,41^ or/and radiative diversification due to ecological opportunities and stepping-stone colonisation of recent Indo-Pacific habitat^25,42–44^. Conversely, we observed a decrease of diversification rates for the Haemulinae during the same period (Fig. 2), whereas an acceleration of the rate of morphological evolution has been reported at the same epoch for this subfamily^45^.

The most recent shift, observed in the diversification rates of Haemulinae, Holocentridae, Labridae, Pomacentridae and Sparidae (Fig. 2), occur during the Quaternary. This period is marked by important climatic variations that constrained the distribution of coral reefs and tropical reef fishes to small refuges^8,46^. This result is not surprising given that the important loss of reef habitat has likely caused numerous extinctions within tropical reef fish lineages^28^.

### Effect of past habitat changes

Our results indicate that diversification rates of only two tropical reef fish clades could show a dependence to reef habitat fragmentation. For Acanthuridae and Holocentridae, the variation of habitat fragmentation has been found to impact the speciation rates. Tropical reefs constitute a habitat with patchily distributed fish populations that are connected through larval dispersal^47,48^. However, those populations can be isolated by soft barriers created by deep-seas or currents which often favours speciation^49^. In consequence, the fragmentation of tropical reef habitats can foster speciation in parapatry^50^ and influence rates of speciation^4^.

Our results indicate that most coral-reef fish groups included in the analysis do not show a dependence of their diversification rates to global reef area, except Haemulinae (Table 3). Although the effect of habitat area has been hypothesized to foster speciation and limit extinction^51,52^ by sustaining large ranges and populations, evidences demonstrate that it is the maintenance of large habitat through long periods, rather than the variation of habitat area, that influences diversification rates^19,53,54^.

**Table 3:** Models tested in the *environmental birth–death* approach.

Model	λ	µ
Model 1	cst	cst
Model 2.1	α + βv(t)	cst
Model 2.2	αe^βv(t)^	cst
Model 3.1	cst	α + βv(t)
Model 3.2	cst	αe^βv(t)^
Model 4.1	α_1_ + β_1_v(t)	α_2_ + β_2_v(t)
Model 4.2	$\alpha_{{1}} {\text{e}}^{{\beta_{1} {\text{v}}({\text{t}})}}$	$\alpha_{2} {\text{e}}^{{\beta_{2} {\text{v}}({\text{t}})}}$

First column describes the model applied on the speciation rate, and second describes the model on extinction rate. The parameters α and β are estimated during the optimization procedure and $v\left(t\right)$ represents the paleo-environmental variable.

### No strong effect of paleo-habitat changes on diversification rates

The general tendency of our results is that we couldn’t find strong evidence of the effect of paleo-habitat change and deep-time geological events on the diversification of most investigated clades. For most clades, using consensus trees and posterior distributions, we were not able to reject our null hypothesis (standard *birth–death* model) against *environmental-birth–death* (Balistoidea, Carangoidea, Chaetodontidae, Labridae, Pomacentridae, Sparidae) and *birth–death-shift* (Balistoidea, Carangoidea, Chaetodontidae, Pomacentridae, Sparidae) models of diversification. This suggest that paleo-habitat changes such habitat fragmentation and total-reef area did not affect diversification rates of those clades at the global scale. This suggests that diversification rates of those clades have been primarily driven by other factors than geological and climatic events. For instance, several lines of evidence suggest that evolution in diet is a major driver of the diversification of Chaetodontidae^24,55^ and Scarinae diversification^56^, and could have influenced the diversification of many other tropical reef fish families^57,58^.

### Robustness of environmental birth–death models to phylogenetic and dating uncertainties

In most cases, results based on consensus trees are not concordant with those based on the posterior distribution of trees. Most of those discrepancies are due to high uncertainties in the results based on posterior distributions. For instance, the consensus tree of Holocentridae fits to an *environmental birth–death* model for which the speciation rate is dependent on the number of patches (Table 2). However, 46% of the trees taken in the posterior distribution of Holocentridae fit an *environmental birth–death* model with a dependence on both speciation and extinction rates. This indicates that the chances that speciation rates of Holocentridae are dependent on habitat fragmentation are high, and that the dependence of extinction rates is not firmly rejected, because of phylogenetic and dating uncertainties. In other cases, the discrepancies are more problematic because the posterior distribution gives overwhelming supports to a hypothesis while the consensus tree supports an alternative hypothesis. For instance, the posterior distribution of Haemulinae gives supports to an *environmental birth–death* model dependent on total reef area at 99%, over a pure *birth–death* model. At the contrary, the consensus tree strongly supports a pure *birth–death* (Table 2). Similarly, *birth–death-shift* analyses can give support to different shifts of diversification between consensus trees and posterior distribution (Fig. 2). Such cases show that our results are only partly robust to phylogenetic and dating uncertainties, and therefore that our findings should be interpreted with caution.

In addition, a single time-calibrated phylogeny can be consistent with an infinite number of time-varying diversification scenarios^59^, which can make the interpretation of estimated diversification rates spurious. In this article, we focussed in testing time-varying models against time-constant models instead of using an exploratory approach^60^. We also focussed on giving priority to the simpler model when the support for alternative hypotheses was not consistent, which means that a rejection of a pure *birth–death* model cannot be attributed to that bias. Finally, we also gave priority to the results coming from posterior distributions over consensus trees as they were including phylogenetic and dating uncertainties and were thus directly interpretable.

## Conclusion and future prospects

Our study suggests that diversification rates of tropical reef fishes is linked to major earth-history events. The periods corresponding to the formation of the Indo-Australian Archipelago and the climatic fluctuations of the Quaternary showed important shifts of diversification rates. However, the use of environmental birth–death models does not provide support for a major role of past habitat changes on the diversification rates variation of tropical reef fishes. To confirm that tendency, future works encompassing a large number of clades with contrasting ecology and life history strategies and focusing on particular regions are needed. Indeed, the fragmentation and surface area of tropical reefs do not only vary through time, but also through space. Consequently, at one point of time, some regions can support highly fragmented and narrow reef habitats, while other regions support continuous and large reef habitats. In this configuration and under the hypothesis of a diversification dependant on habitat dynamics, species from the same clade that are distributed in different regions should show antagonistic diversification modes^26,27,30,45,61–63^.

## Methods

### Paleo-environment

We reconstructed the potential habitat of coral reef fish since the Cenozoic using a model of synthetic paleobathymetry coupled with reef-forming coral fossil records^4^. The Paleobathymetry model is based on global ocean paleo-age grids^64^ merged with paleocoastline distributions for continental areas. It outlines the absolute position of continents over time and the associated distribution and residence time of shallow epicontinental seas^65^. Reef-forming coral species can only develop at warm water temperature (> 25 °C, Kleypas and Mcmanus^66^). It is then straightforward to identify the past latitudinal limits of tropical habitat based on the fossil record. We obtained 1 map, for each million year since the Cenozoic, of tropical shallow marine habitat at a 1° resolution. In order to use metrics based on habitat area, we projected each map using a Behrmann cylindrical equal-area projection, which has a low shape distortion in low latitudes^67^. For each map we calculated the fragmentation as the number of patches of available habitat (number of independent patches of habitat cells surrounded by non-habitat cells) and the total area (area) of available habitat in Km^2^ (Fig. 1), which allowed us to evaluate the effect of coral reef habitat fragmentation and area independently.

### Fish phylogenies

We gathered 9 recently built, time-calibrated, phylogenies of coral reef fishes from different sources (Acanthuridae, Balistoidea, Carangoidea, Chaetodontidae, Haemulinae, Holocentridae, Labridae, Pomacentridae, Sparidae). As their taxonomic rank isn’t homogeneous (from Superfamily to subfamily), we will simply refer as taxa for all of those Superfamilies/families/subfamilies. Although not identical, approaches used for phylogenetic reconstruction and divergence times estimation of each clades were similar. For each taxon, phylogenies were explored using Bayesian algorithms and both nuclear and mitochondrial genes and divergence times were estimated using Bayesian relaxed clock models with fossil calibrations (Table 4). To limit and assess bias due to phylogenetic and dating uncertainties, we only selected phylogenies that had a phylogenetic sampling higher than 50%, as likelihood surface of complete phylogenies differs qualitatively little compared with the surface at 50% sampling, using the sampling fraction method implemented in diversification models we use^68^.

**Table 4:** Coral-reef fish phylogenies included in the analysis.

Tree	Number of species	Sampling	Mean size (cm)	Reproduction	Reference
Acanthuridae	63	0.78	40.12	Scatterers	Sorenson et al.^26^
Balistoidea	80	0.54	29.09	Nesters	McCord and Westneat^61^
Carangoidea	131	0.85	70.02	Scatterers	Santini and Carnevale^62^
Chaetodontidae	100	0.78	17.19	Scatterers	Gaboriau el al.^30^
Haemulinae	50	0.85	44.78	Scatterers	Price et al.^42^
Holocentridae	42	0.51	23.67	Scatterers	Dornburg et al.^27^
Labridae	268	0.57	26.24	Scatterers	Gaboriau el at.^30^
Pomacentridae	225	0.6	11.31	Nesters	Gaboriau et al.^30^
Sparidae	91	0.75	55.33	Scatterers	Santini et al.^63^

The Number of species is the number of species present in the phylogenetic tree. The Sampling column represents the phylogenetic sampling.

### Diversification analyses

We first tested the hypothesis that the diversification rates of those taxa vary through time in conjunction with selected major paleo-environment events, using a likelihood maximization of a *birth–death-shift* process implemented in the R package *TreePar*^34^. This method allows us to test for the presence of one or several shifts in diversification rate by comparing the likelihood of the chronogram under different *birth–death-shift* models. Given a fixed number of shifts and the phylogenetic sampling, the optimisation procedure simultaneously estimates the date(s) of the shift(s), and the diversification rate during each delineated period of time. For each taxon we tested for the presence of a shift in diversification rate every 5my to identify congruence between environmental or geological changes and shifts in diversification rate. To select the optimal number of shifts we compared the corrected Akaike information criterion (AICc) of each model. The model with the lower AICc was considered the best and all the models for which the difference between the lower AICc and their AICc ($\Delta AICc$) is above two are considered significantly less explicative. This method relies on branching times and is thus really sensitive to phylogenetic uncertainties and node age estimations. We used the sampling fraction approach to compensate the effect of incomplete taxon sampling (ITS) assuming that ITS was homogeneous along the trees. As posterior distributions of chronogram reconstructions are informative of both dating and phylogenetic uncertainties, we compared the results of *birth–death-shift* models applied to 1000 trees from each posterior distribution of the 9 taxa. For each tree, we selected the model (number of shift) that minimized the AICc. We were then able to estimate which is the most frequently selected number of shifts for each taxon. Based on those results, we estimated the median and the first and ninth decile of the estimated diversification rate distribution, for each taxon, every 100,000 years.

Then, we tested for the effect of the paleo-environment on diversification rate using a likelihood maximization of an environmental birth–death process. Those models are classic birth–death models that allow λ (speciation rate) and μ (extinction rate) to change through time depending on a paleo-environmental variable^15^. They are implemented in the R package RPANDA^69^. Paleo-environmental variables such as habitat fragmentation and paleo-temperature can have an influence on both speciation and extinction. To make the distinction, we built independent models where the paleo-environmental variable respectively influenced λ, μ and both λ and μ. We only tested linear and exponential relations between speciation/extinction and the environmental variables and compared all those models with a simple birth–death model, which led us to a total of seven independent models (Table 3) for each tree. This method also relies on branching times and is thus really sensible to phylogenetic uncertainties and node age estimations. We took this into account by applying those seven models to 1000 trees from the posterior distributions of each phylogeny. For each tree we selected the model with the lower AICc and all the models that have a ΔAICc lower than 2. We also used the sampling fraction approach to compensate the effect of incomplete taxon sampling (ITS) assuming that ITS was homogeneous along the trees. With this approach, we tested for the effect of paleo-habitat variables (number of patches, total area) on speciation and extinction rates through time.

## Supplementary Information

Supplementary Information.

## References

[1] Barnosky AD (2011). Has the Earth’s sixth mass extinction already arrived?. Nature.

[2] Zaffos A, Finnegan S, Peters SE (2017). Plate tectonic regulation of global marine animal diversity. Proc. Natl. Acad. Sci..

[3] Claramunt S, Cracraft J (2015). A new time tree reveals Earth historys imprint on the evolution of modern birds. Sci. Adv..

[4] Leprieur F, Descombes P, Gaboriau T, Cowman PF, Parravicini V (2016). Plate tectonics drive tropical reef biodiversity dynamics. Nat. Commun..

[5] Ficetola GF, Mazel F, Thuiller W (2017). Global determinants of zoogeographical boundaries. Nat. Ecol. Evol..

[6] Mazel F (2017). Global patterns of β-diversity along the phylogenetic time-scale: The role of climate and plate tectonics. Glob. Ecol. Biogeogr..

[7] Hofreiter M, Stewart J (2009). Ecological change, range fluctuations and population dynamics during the pleistocene. Curr. Biol..

[8] Pellissier L (2014). Quaternary coral reef refugia preserved fish diversity. Science.

[9] Jaramillo C (2010). Effects of rapid global warming at the paleocene-eocene boundary on neotropical vegetation. Science.

[10] Svenning J-C, Eiserhardt WL, Normand S, Ordonez A, Sandel B (2015). The influence of paleoclimate on present-day patterns in biodiversity and ecosystems. Annu. Rev. Ecol. Evol. Syst..

[11] Steeman ME (2009). Radiation of extant cetaceans driven by restructuring of the oceans. Syst. Biol..

[12] Antonelli A, Sanmartín I (2011). Mass Extinction, gradual cooling, or rapid radiation? reconstructing the spatiotemporal evolution of the ancient angiosperm genus hedyosmum (Chloranthaceae) using empirical and simulated approaches. Syst. Biol..

[13] Nee S, May RM, Harvey PH (1994). The reconstructed evolutionary process. Philos. Trans. R. Soc. Lond. B.

[14] Morlon H, Parsons TL, Plotkin JB (2011). From the cover: Reconciling molecular phylogenies with the fossil record. Proc. Natl. Acad. Sci..

[15] Condamine FL, Rolland J, Morlon H (2013). Macroevolutionary perspectives to environmental change. Ecol. Lett..

[16] Condamine FL (2015). Deciphering the evolution of birdwing butterflies 150 years after Alfred Russel Wallace Deciphering the evolution of birdwing butterflies 150 years after. Sci. Rep..

[17] Lagomarsino LP, Condamine FL, Antonelli A, Mulch A, Davis CC (2016). The abiotic and biotic drivers of rapid diversification in Andean bellflowers (Campanulaceae). New Phytol..

[18] Rolland J, Condamine FL (2019). The contribution of temperature and continental fragmentation to amphibian diversification. J. Biogeogr..

[19] Gaboriau T (2019). Ecological constraints coupled with deep-time habitat dynamics predict the latitudinal diversity gradient in reef fishes. Proc. R. Soc. B Biol. Sci..

[20] Descombes P (2017). Linking species diversification to palaeo-environmental changes: A process-based modelling approach. Glob. Ecol. Biogeogr..

[21] Rangel TF (2018). Modeling the ecology and evolution of biodiversity: Biogeographical cradles, museums, and graves. Science.

[22] Pontarp M (2019). The latitudinal diversity gradient: Novel understanding through mechanistic eco-evolutionary models. Trends Ecol. Evol..

[23] Cowman PF (2014). Historical factors that have shaped the evolution of tropical reef fishes: A review of phylogenies, biogeography, and remaining questions. Front. Genet..

[24] Bellwood DR (2010). Evolutionary history of the butterflyfishes (f: Chaetodontidae) and the rise of coral feeding fishes. J. Evol. Biol..

[25] Cowman PF, Bellwood DR (2011). Coral reefs as drivers of cladogenesis: Expanding coral reefs, cryptic extinction events, and the development of biodiversity hotspots. J. Evol. Biol..

[26] Sorenson L, Santini F, Carnevale G, Alfaro ME (2013). A multi-locus timetree of surgeonfishes (Acanthuridae, Percomorpha), with revised family taxonomy. Mol. Phylogenet. Evol..

[27] Dornburg A, Moore J, Beaulieu JM, Eytan RI, Near TJ (2015). The impact of shifts in marine biodiversity hotspots on patterns of range evolution: Evidence from the Holocentridae (squirrelfishes and soldierfishes). Evolution.

[28] Cowman PF, Bellwood DR (2013). The historical biogeography of coral reef fishes: Global patterns of origination and dispersal. J. Biogeogr..

[29] Lohman DJ (2011). Biogeography of the Indo-Australian archipelago. Annu. Rev. Ecol. Evol. Syst..

[30] Gaboriau T, Leprieur F, Mouillot D, Hubert N (2017). Influence of the geography of speciation on current patterns of coral reef fish biodiversity across the Indo-Pacific. Ecography.

[31] McManus JW (1985). Marine speciation, tectonics and sea- level changes in Southeast Asia. Proc. Fifth Int. Coral Reef.

[32] Potts DC (1985). Sea-level fluctuations and speciation in Scleractinia. Proc. Fifth Int. Coral Reef.

[33] Hou Z, Li S (2017). Tethyan changes shaped aquatic diversification. Biol. Rev..

[34] Stadler T (2011). Mammalian phylogeny reveals recent diversification rate shifts. Proc. Natl. Acad. Sci. USA.

[35] Bellwood, D. R. & Wainwright, P. C. The history and biogeography of Fishes on Coral Reefs. in *Coral Reef Fishes, Dynamics and Diversity in a Complex Ecosystem*, 5–32 (2002).

[36] Williams ST, Duda TF (2008). Did tectonic activity stimulate Oligo-Miocene speciation in the Indo-West Pacific?. Evolution.

[37] Renema W (2008). Hopping hotspots: Global shifts in marine biodiversity. Science.

[38] Tea Y-K (2021). Phylogenomic analysis of concatenated ultraconserved elements reveals the recent evolutionary radiation of the fairy wrasses (teleostei: labridae: cirrhilabrus). Syst. Biol..

[39] Hall R (2009). Southeast Asia’s changing palaeogeography. Blumea J. Plant Taxon. Plant Geogr..

[40] Keith SA, Baird AH, Hughes TP, Madin JS, Connolly SR (2013). Faunal breaks and species composition of Indo-Pacific corals: The role of plate tectonics, environment and habitat distribution. Proc. Biol. Sci..

[41] Cowman PF, Bellwood DR (2013). Vicariance across major marine biogeographic barriers: Temporal concordance and the relative intensity of hard versus soft barriers. Proc. Biol. Sci..

[42] Price SA, Claverie T, Near TJ, Wainwright PC (2015). Phylogenetic insights into the history and diversification of fishes on reefs. Coral Reefs.

[43] Bellwood DR, Goatley CHR, Bellwood O (2017). The evolution of fishes and corals on reefs: Form, function and interdependence. Biol. Rev..

[44] Bowen BW, Rocha LA, Toonen RJ, Karl SA (2013). The origins of tropical marine biodiversity. Trends Ecol. Evol..

[45] Price SA, Tavera JJ, Near TJ, Wainwright PC (2012). Elevated rates of morphological and functional diversification in reef-dwelling haemulid fishes. Evolution.

[46] Kiessling W, Simpson C, Beck B, Mewis H, Pandolfi JM (2012). Equatorial decline of reef corals during the last Pleistocene interglacial. Proc. Natl. Acad. Sci. USA..

[47] Floeter SR (2007). Atlantic reef fish biogeography and evolution. J. Biogeogr..

[48] Riginos C, Buckley YM, Blomberg SP, Treml EA (2014). Dispersal capacity predicts both population genetic structure and species richness in reef fishes. Am. Nat..

[49] Rocha LA, Bowen BW (2008). Speciation in coral-reef fishes. J. Fish Biol..

[50] Tedesco PA, Paradis E, EvEque CL, Hugueny B (2016). Explaining global-scale diversification patterns in actinopterygian fishes. J. Biogeogr..

[51] Rosenzweig ML (1995). Species Diversity in Space and Time.

[52] Kisel Y, Barraclough TG (2010). Speciation has a spatial scale that depends on levels of gene flow. Am. Nat..

[53] Fine PVA, Ree RH (2006). Evidence for a time-integrated species-area effect on the latitudinal gradient in tree diversity. Am. Nat..

[54] Jetz W, Fine PVA (2012). Global gradients in vertebrate diversity predicted by historical area-productivity dynamics and contemporary environment. PLoS Biol..

[55] Konow N, Price S, Abom R, Bellwood D, Wainwright P (2017). Decoupled diversification dynamics of feeding morphology following a major functional innovation in marine butterflyfishes. Proc. Biol. Sci..

[56] Clements KD, German DP, Piché J, Tribollet A, Choat JH (2017). Integrating ecological roles and trophic diversification on coral reefs: Multiple lines of evidence identify parrotfishes as microphages. Biol. J. Linn. Soc..

[57] Lobato FL (2014). Diet and diversification in the evolution of coral reef fishes. PLoS ONE.

[58] Siqueira AC, Morais RA, Bellwood DR, Cowman PF (2020). Trophic innovations fuel reef fish diversification. Nat. Commun..

[59] Louca S, Pennell MW (2020). Extant timetrees are consistent with a myriad of diversification histories. Nature.

[60] Morlon, H., Hartig, F. & Robin, S. Prior hypotheses or regularization allow inference of diversification histories from extant timetrees. *bioRxiv* (2020).

[61] McCord CL, Westneat MW (2016). Phylogenetic relationships and the evolution of BMP4 in triggerfishes and filefishes (Balistoidea). Mol. Phylogenet. Evol..

[62] Santini F, Carnevale G (2015). First multilocus and densely sampled timetree of trevallies, pompanos and allies (Carangoidei, Percomorpha) suggests a Cretaceous origin and Eocene radiation of a major clade of piscivores. Mol. Phylogenet. Evol..

[63] Santini F, Carnevale G, Sorenson L (2014). First multi-locus timetree of seabreams and porgies (Percomorpha: Sparidae). Ital. J. Zool..

[64] Müller RD, Sdrolias M, Gaina C, Steinberger B, Heine C (2008). Long-term sea-level fluctuations driven by ocean basin dynamics. Science.

[65] Heine C, Yeo LG, Müller RD (2015). Evaluating global paleoshoreline models for the Cretaceous and Cenozoic. Aust. J. Earth Sci..

[66] Kleypas JA, Mcmanus JW (1999). Environmental Limits to Coral Reef Development : Where Do We Draw the Line ?. Am. Zool..

[67] Bugayevskiy LM, Snyder JP (1995). Map Projections: A Reference Manual.

[68] Chang J, Rabosky DL, Alfaro ME (2020). Estimating diversification rates on incompletely sampled phylogenies: Theoretical concerns and practical solutions. Syst. Biol..

[69] Morlon H (2016). RPANDA: An R package for macroevolutionary analyses on phylogenetic trees. Methods Ecol. Evol..

