# Supplementary material for: Influence of historical changes in tropical reef habitat on the diversification of coral reef fishes
Source: Sci Rep. 2021 Oct 20;11:20731. doi: 10.1038/s41598-021-00049-4 (PMC8528860; doi:10.1038/s41598-021-00049-4)
Supplement: Supplementary file 1 — Supplementary Information. [file 41598_2021_49_MOESM1_ESM.pdf]

Influence of historical changes in tropical reef habitat on the diversification of coral reef fishes

Fabien Leprieur[1,2], Loic Pellissier[3,4], David Mouillot[1], Théo Gaboriau[5*]

[1] UMR MARBEC (CNRS, IRD, IFREMER, UM), Université de Montpellier, Place Eugène Bataillon, 34095 Montpellier Cedex 5, France.

[2] Institut Universitaire de France, Paris, France.

[3] Landscape Ecology, Institute of Terrestrial Ecosystems, ETH Zürich, 8092 Zürich, Switzerland

[4] Swiss Federal Research Institute WSL, 8903 Birmensdorf, Switzerland.

[5] Department of Computational Biology University of Lausanne, 1015, Lausanne, Switzerland.

* Corresponding Author: Théo Gaboriau, Department of Computational Biology University of Lausanne, 1015, Lausanne, Switzerland. Email : theo.gaboriau@unil.ch

SUPPLEMMENTARY RESULTS

Effect of paleo-temperature. As variation of paleo-temperatures strongly affected the extent of coral habitat trough time (Kiessling et al. 2012; Pellissier et al. 2014), we expect that paelo-temperature could also have influenced diversification rates of coral-reef fishes. We tested the association between paleo-temperature (Zachos, et al. 2008) and diversification rates with the *environmental birth-death* model following the same approach described in the methods. This analysis rendered contrasted results. The temperature dependant models are never fully rejected by the AICc comparison. For Balistoidea, Carangoidea and Labridae, the best fitting model to the consensus tree is the pure *birth-death* model, but temperature dependant models have a $\Delta AICc$ lower than two. All the other consensus trees are better explained by a temperature dependant model (Tab. 4). Overall, the temperature dependant model was selected in 56.5% of all investigated trees. However, the influence of the paleo-temperature was found to be different among those taxa. This variable was found to influence the speciation rate of the Sparidae, the extinction rate of Acanthuridae, Chaetodontidae and Pomacentridae and both extinction and speciation rates of Haemulinae, Holocentridae and Scarinae. The pure *birth-death* model was significantly outperformed by the temperature dependant model only on the prediction of the Acanthuridae, Haemulinae, Holocentridae and Scarinae consensus trees (Tab. 4). For the temperature, the results based on the posterior distributions of trees are not consistent with those based on the consensus trees. The pure birth-death model is the most frequently selected model in Chaetodontidae, Pomacentridae and Sparidae posterior distributions of trees while the temperature dependant model influencing only speciation is the most frequently selected in Haemulinae, Holocentridae and Labrinae posterior distributions of trees. The influence of paleo-temperature on diversification rates of coral reef fish can be due to many factors such as kinetic effects (Gillooly et al. 2005; Allen et al. 2006) or loss of habitat (Kiessling et al. 2012; Pellissier et al. 2014).

BIBLIOGRPAHY

Allen, A. P., J. F. Gillooly, V. M. Savage, and J. H. Brown. 2006. "Kinetic Effects of Temperature on Rates of Genetic Divergence and Speciation." *Proceedings of the National Academy of Sciences* 103 (24): 9130–35. doi:10.1073/pnas.0603587103.

Gillooly, J. F., A. P. Allen, G. B. West, and J. H. Brown. 2005. "The Rate of DNA Evolution: Effects of Body Size and Temperature on the Molecular Clock." *Proceedings of the National Academy of Sciences* 102 (1): 140–45. doi:10.1073/pnas.0407735101.

Kiessling, Wolfgang, Carl Simpson, Brian Beck, Heike Mewis, and John M Pandolfi. 2012. "Equatorial Decline of Reef Corals during the Last Pleistocene Interglacial." *Proceedings of the National Academy of Sciences of the United States of America* 109 (52): 21378–83. doi:10.1073/pnas.1214037110.

Pellissier, Loïc, Fabien Leprieur, Valeriano Parravicini, Peter F Cowman, Michel Kulbicki, Glenn Litsios, Steffen M Olsen, Mary S Wisz, David R Bellwood, and David Mouillot. 2014. "Quaternary Coral Reef Refugia Preserved Fish Diversity." *Science (New York, N.Y.)* 344 (6187): 1016–19. doi:10.1126/science.1249853.

Zachos, James C., Gerald R. Dickens, and Richard E. Zeebe. 2008. "An Early Cenozoic Perspective on Greenhouse Warming and Carbon-Cycle Dynamics." *Nature* 451 (7176): 279–83. doi:10.1038/nature06588.

SUPPLEMNTARY FIGURES

Table S1. Results of the birth-death-shift models' comparison for the consensus tree of Acanthuridae. The models are sorted by $AICc$ in ascending order. We denote the $AICc$, $\Delta AICc$ and the model parameters (d: diversification rates, shift: shift date).

Nb	AICc	ΔAICc	d_0	shift$_1$	d_1	shift$_2$	d_2	shift$_3$	d_3	shift$_4$	d_4	shift$_5$	d_5	shift$_6$	d_6	shift$_7$	d_7	shift$_8$	d_8	shift$_9$	d_9	shift$_{10}$	d_{10}
1	414.37	0.00	0.10	23.89	0.02	-	-	-	-	-	-	-	-	-	-	-	-	-	-	-	-	-	-
2	415.71	1.34	0.07	3.89	0.12	23.89	0.02	-	-	-	-	-	-	-	-	-	-	-	-	-	-	-	-
3	417.37	3.00	0.07	3.89	0.12	23.89	0.00	33.89	0.04	-	-	-	-	-	-	-	-	-	-	-	-	-	-
0	417.87	3.50	0.08	-	-	-	-	-	-	-	-	-	-	-	-	-	-	-	-	-	-	-	-
4	419.46	5.09	0.08	3.89	0.12	23.89	0.00	33.89	0.06	43.89	0.00	-	-	-	-	-	-	-	-	-	-	-	-
5	423.37	9.00	0.07	3.89	0.12	18.89	0.12	23.89	0.00	33.89	0.06	43.89	0.00	-	-	-	-	-	-	-	-	-	-
6	426.81	12.44	0.07	3.89	0.12	18.89	0.00	23.89	0.00	33.89	0.08	43.89	0.00	53.89	0.12	-	-	-	-	-	-	-	-
7	430.72	16.35	0.07	3.89	0.11	18.89	0.00	23.89	0.00	28.89	0.00	33.89	0.10	43.89	0.00	53.89	0.14	-	-	-	-	-	-
8	434.70	20.33	0.07	3.89	0.11	18.89	0.00	23.89	0.00	28.89	0.00	33.89	0.10	38.89	0.12	43.89	0.00	53.89	0.15	-	-	-	-
9	438.70	24.33	0.07	3.89	0.11	18.89	0.00	23.89	0.00	28.89	0.00	33.89	0.10	38.89	0.12	43.89	0.00	48.89	0.00	53.89	0.02	-	-
10	445.89	31.52	0.07	3.89	0.11	13.89	0.02	18.89	0.15	23.89	0.01	28.89	0.00	33.89	0.07	38.89	0.07	43.89	0.00	48.89	0.01	53.89	0.03

Table S2. Results of the birth-death-shift models' comparison for the consensus tree of Balistoidae. The models are sorted by $AICc$ in ascending order. We denote the $AICc$, $\Delta AICc$ and the model parameters (d: diversification rates, shift: shift date).

Nb	AICc	ΔAICc	d_0	$shift_1$	d_1	$shift_2$	d_2	$shift_3$	d_3	$shift_4$	d_4	$shift_5$	d_5	$shift_6$	d_6	$shift_7$	d_7	$shift_8$	d_8	$shift_9$	d_9	$shift_{10}$	d_{10}
1	573.36	0.00	-0.03	11.54	0.06	-	-	-	-	-	-	-	-	-	-	-	-	-	-	-	-	-	-
2	574.42	1.06	-0.03	11.54	0.08	41.54	0.00	-	-	-	-	-	-	-	-	-	-	-	-	-	-	-	-
3	576.64	3.29	0.00	6.54	0.01	11.54	0.11	41.54	0.00	-	-	-	-	-	-	-	-	-	-	-	-	-	-
0	578.33	4.97	0.08	-	-	-	-	-	-	-	-	-	-	-	-	-	-	-	-	-	-	-	-
4	579.46	6.11	0.00	6.54	0.01	11.54	0.11	26.54	0.08	41.54	0.00	-	-	-	-	-	-	-	-	-	-	-	-
5	582.62	9.26	0.00	6.54	0.01	11.54	0.12	26.54	0.04	31.54	0.07	41.54	0.00	-	-	-	-	-	-	-	-	-	-
6	585.87	12.51	0.00	1.54	0.03	6.54	0.03	11.54	0.11	26.54	0.03	31.54	0.06	41.54	0.00	-	-	-	-	-	-	-	-
7	589.52	16.16	0.00	1.54	0.04	6.54	0.02	11.54	0.12	16.54	0.09	26.54	0.01	31.54	-0.05	41.54	0.00	-	-	-	-	-	-
8	593.18	19.83	0.00	1.54	0.04	6.54	0.03	11.54	0.07	16.54	0.09	26.54	0.01	31.54	-0.05	36.54	-0.04	41.54	0.00	-	-	-	-
9	596.92	23.57	0.00	1.54	0.05	6.54	0.03	11.54	0.07	16.54	0.08	21.54	0.04	26.54	-0.05	31.54	0.00	36.54	-0.10	41.54	0.00	-	-
10	599.30	25.95	0.00	1.54	0.11	6.54	0.01	11.54	0.02	16.54	0.05	21.54	0.07	26.54	-0.59	31.54	0.00	36.54	-0.42	41.54	0.00	51.54	0.06

Table S3. Results of the birth-death-shift models' comparison for the consensus tree of Carangoidae. The models are sorted by $AICc$ in ascending order. We denote the $AICc$, $\Delta AICc$ and the model parameters (d: diversification rates, shift: shift date).

Nb	AICc	ΔAICc	d_0	$shift_1$	d_1	$shift_2$	d_2	$shift_3$	d_3	$shift_4$	d_4	$shift_5$	d_5	$shift_6$	d_6	$shift_7$	d_7	$shift_8$	d_8	$shift_9$	d_9	$shift_{10}$	d_{10}	$shift_{11}$	d_{11}	$shift_{12}$	d_{12}	$shift_{13}$	d_{13}	$shift_{14}$	d_{14}	$shift_{15}$	d_{15}	$shift_{16}$	d_{16}	$shift_{17}$	d_{17}
0	1021.85	0.00	0.05	-	-	-	-	-	-	-	-	-	-	-	-	-	-	-	-	-	-	-	-	-	-	-	-	-	-	-	-	-	-	-	-	-	-
1	1022.86	1.01	0.05	17.26	0.03	-	-	-	-	-	-	-	-	-	-	-	-	-	-	-	-	-	-	-	-	-	-	-	-	-	-	-	-	-	-	-	-
2	1025.43	3.57	0.05	12.26	0.06	17.26	0.03	-	-	-	-	-	-	-	-	-	-	-	-	-	-	-	-	-	-	-	-	-	-	-	-	-	-	-	-	-	-
3	1027.89	6.03	0.05	12.26	0.06	17.26	0.07	32.26	0.03	-	-	-	-	-	-	-	-	-	-	-	-	-	-	-	-	-	-	-	-	-	-	-	-	-	-	-	-
4	1030.72	8.86	0.05	12.26	0.05	17.26	0.08	32.26	0.02	67.26	0.08	-	-	-	-	-	-	-	-	-	-	-	-	-	-	-	-	-	-	-	-	-	-	-	-	-	-
5	1033.81	11.95	0.05	12.26	0.04	17.26	0.07	32.26	0.00	67.26	0.19	72.26	0.06	-	-	-	-	-	-	-	-	-	-	-	-	-	-	-	-	-	-	-	-	-	-	-	-
6	1037.08	15.23	0.05	12.26	0.04	17.26	0.08	32.26	0.00	67.26	0.18	72.26	0.04	82.26	0.00	-	-	-	-	-	-	-	-	-	-	-	-	-	-	-	-	-	-	-	-	-	-
7	1039.72	17.87	0.05	12.26	0.04	17.26	0.07	32.26	0.00	67.26	0.19	72.26	0.07	77.26	0.00	82.26	0.00	-	-	-	-	-	-	-	-	-	-	-	-	-	-	-	-	-	-	-	-
8	1043.67	21.82	0.05	12.26	0.03	17.26	0.08	32.26	0.00	67.26	0.20	72.26	0.05	77.26	0.00	82.26	0.00	87.26	0.04	-	-	-	-	-	-	-	-	-	-	-	-	-	-	-	-	-	-
9	1050.65	28.80	0.05	12.26	0.03	17.26	0.08	32.26	0.00	62.26	0.18	67.26	0.04	72.26	0.08	77.26	0.00	82.26	0.00	87.26	0.04	-	-	-	-	-	-	-	-	-	-	-	-	-	-	-	-
11	1054.73	32.87	0.05	12.26	0.03	17.26	0.07	27.26	0.00	32.26	0.06	57.26	0.00	62.26	0.03	67.26	0.02	72.26	0.01	77.26	0.00	82.26	0.00	87.26	0.01	-	-	-	-	-	-	-	-	-	-	-	-
10	1055.00	33.14	0.05	12.26	0.03	17.26	0.06	27.26	0.00	32.26	0.05	62.26	0.02	67.26	0.05	72.26	0.05	77.26	0.00	82.26	0.01	87.26	0.04	-	-	-	-	-	-	-	-	-	-	-	-	-	-
12	1057.62	35.76	0.05	12.26	0.03	17.26	0.06	27.26	0.00	32.26	0.08	37.26	0.01	57.26	0.00	62.26	0.01	67.26	0.02	72.26	0.00	77.26	0.00	82.26	0.01	87.26	0.02	-	-	-	-	-	-	-	-	-	-
13	1060.81	38.96	0.05	12.26	0.03	17.26	0.08	22.26	0.00	27.26	0.12	32.26	0.01	37.26	0.03	57.26	0.00	62.26	0.01	67.26	0.00	72.26	0.01	77.26	0.00	82.26	0.01	87.26	0.04	-	-	-	-	-	-	-	-
14	1064.51	42.65	0.05	12.26	0.03	17.26	0.08	22.26	0.00	27.26	0.12	32.26	0.01	37.26	0.03	42.26	0.01	57.26	0.00	62.26	0.00	67.26	0.01	72.26	0.00	77.26	0.00	82.26	0.01	87.26	0.06	-	-	-	-	-	-
15	1068.03	46.18	0.05	12.26	0.03	17.26	0.09	22.26	0.00	27.26	0.11	32.26	0.01	37.26	0.02	42.26	0.00	52.26	0.01	57.26	0.00	62.26	0.00	67.26	0.00	72.26	0.00	77.26	0.00	82.26	0.00	87.26	0.15	-	-	-	-
16	1072.07	50.22	0.05	12.26	0.03	17.26	0.08	22.26	0.00	27.26	0.11	32.26	0.01	37.26	0.02	42.26	0.00	47.26	0.00	52.26	0.00	57.26	0.00	62.26	0.00	67.26	0.01	72.26	0.01	77.26	0.00	82.26	0.00	87.26	0.15	-	-
17	1078.81	56.95	0.04	7.26	0.02	12.26	0.06	17.26	0.00	22.26	0.08	27.26	0.02	32.26	0.03	37.26	0.00	42.26	0.01	47.26	0.00	52.26	0.03	57.26	0.00	62.26	0.01	67.26	0.04	72.26	0.01	77.26	0.00	82.26	0.00	87.26	0.20

Table S4. Results of the birth-death-shift models' comparison for the consensus tree of Chaetodontidae. The models are sorted by $AICc$ in ascending order. We denote the $AICc$, $\Delta AICc$ and the model parameters (d: diversification rates, shift: shift date).

Nb	AICc	ΔAICc	d_0	$shift_1$	d_1	$shift_2$	d_2	$shift_3$	d_3	$shift_4$	d_4	$shift_5$	d_5	$shift_6$	d_6
0	548.19	0.00	0.13	-	-	-	-	-	-	-	-	-	-	-	-
1	549.42	1.23	0.14	26.72	0.00	-	-	-	-	-	-	-	-	-	-
2	552.18	3.99	0.14	21.72	-0.16	26.72	0.01	-	-	-	-	-	-	-	-
3	552.86	4.67	0.16	16.72	-0.05	21.72	-0.20	26.72	0.00	-	-	-	-	-	-
4	556.52	8.33	0.16	11.72	-0.08	16.72	-0.83	21.72	-0.11	26.72	0.00	-	-	-	-
5	560.40	12.21	0.16	11.72	-0.08	16.72	-0.82	21.72	-0.11	26.72	0.00	31.72	3.46	-	-
6	570.19	22.00	0.18	6.72	-0.02	11.72	0.00	16.72	-0.01	21.72	0.46	26.72	0.01	31.72	3.27

Table S5. Results of the birth-death-shift models' comparison for the consensus tree of Haemulinae. The models are sorted by $AICc$ in ascending order. We denote the $AICc$, $\Delta AICc$ and the model parameters (d: diversification rates, shift: shift date).

Nb	AICc	ΔAICc	d_0	$shift_1$	d_1	$shift_2$	d_2	$shift_3$	d_3	$shift_4$	d_4	$shift_5$	d_5	$shift_6$	d_6	$shift_7$	d_7	$shift_8$	d_8	$shift_9$	d_9
1	379.94	0.00	0.02	6.23	0.06	-	-	-	-	-	-	-	-	-	-	-	-	-	-	-	-
2	380.98	1.04	0.02	6.23	0.00	26.23	0.12	-	-	-	-	-	-	-	-	-	-	-	-	-	-
3	381.86	1.92	0.00	6.23	0.00	26.23	-0.11	31.23	0.25	-	-	-	-	-	-	-	-	-	-	-	-
4	383.02	3.07	0.00	6.23	0.00	16.23	-0.12	26.23	-0.48	31.23	0.24	-	-	-	-	-	-	-	-	-	-
0	385.20	5.26	0.05	-	-	-	-	-	-	-	-	-	-	-	-	-	-	-	-	-	-
5	385.43	5.49	-0.01	6.23	0.00	16.23	-0.23	21.23	0.00	26.23	-0.50	31.23	0.24	-	-	-	-	-	-	-	-
6	387.83	7.88	0.00	6.23	0.03	16.23	-0.34	21.23	0.00	26.23	-0.62	31.23	0.48	36.23	0.31	-	-	-	-	-	-
7	389.88	9.94	0.00	6.23	0.03	16.23	-0.34	21.23	0.00	26.23	-0.82	31.23	0.48	36.23	0.64	41.23	0.00	-	-	-	-
8	393.37	13.43	0.00	6.23	0.03	16.23	-0.46	21.23	0.00	26.23	-0.79	31.23	0.41	36.23	0.80	41.23	0.00	46.23	0.03	-	-
9	416.36	36.42	0.00	6.23	0.02	11.23	-0.40	16.23	-0.11	21.23	-0.58	26.23	0.36	31.23	0.65	36.23	0.02	41.23	0.00	46.23	0.12

Table S6. Results of the birth-death-shift models' comparison for the consensus tree of Holocentridae. The models are sorted by $AICc$ in ascending order. We denote the $AICc$, $\Delta AICc$ and the model parameters (d: diversification rates, shift: shift date).

Nb	AICc	ΔAICc	d_0	$shift_1$	d_1	$shift_2$	d_2	$shift_3$	d_3	$shift_4$	d_4	$shift_5$	d_5	$shift_6$	d_6	$shift_7$	d_7
2	267.72	0.00	0.07	4.63	0.14	24.63	0.00	-	-	-	-	-	-	-	-	-	-
1	268.47	0.74	0.10	24.63	0.00	-	-	-	-	-	-	-	-	-	-	-	-
0	270.51	2.79	0.10	-	-	-	-	-	-	-	-	-	-	-	-	-	-
3	271.08	3.36	0.07	4.63	0.13	19.63	0.07	24.63	0.00	-	-	-	-	-	-	-	-
4	274.63	6.91	0.07	4.63	0.12	14.63	0.19	19.63	0.07	24.63	0.00	-	-	-	-	-	-
5	277.47	9.75	0.07	4.63	0.03	9.63	0.17	14.63	0.27	19.63	0.09	24.63	0.00	-	-	-	-
6	281.24	13.52	0.07	4.63	0.03	9.63	0.16	14.63	0.25	19.63	0.08	24.63	0.00	39.63	0.04	-	-
7	285.24	17.52	0.07	4.63	0.03	9.63	0.16	14.63	0.25	19.63	0.08	24.63	0.00	29.63	0.00	39.63	0.06

Table S7. Results of the birth-death-shift models' comparison for the consensus tree of Labrinae. The models are sorted by $AICc$ in ascending order. We denote the $AICc$, $\Delta AICc$ and the model parameters (d: diversification rates, shift: shift date).

Nb	AICc	ΔAICc	d_0	$shift_1$	d_1	$shift_2$	d_2	$shift_3$	d_3	$shift_4$	d_4	$shift_5$	d_5	$shift_6$	d_6	$shift_7$	d_7	$shift_8$	d_8	$shift_9$	d_9	$shift_{10}$	d_{10}	$shift_{11}$	d_{11}	$shift_{12}$	d_{12}
1	2477.15	0.00	0.05	2.64	0.08	-	-	-	-	-	-	-	-	-	-	-	-	-	-	-	-	-	-	-	-	-	-
2	2480.15	3.00	0.04	2.64	0.07	22.64	0.07	-	-	-	-	-	-	-	-	-	-	-	-	-	-	-	-	-	-	-	-
3	2483.00	5.85	0.04	2.64	0.07	22.64	0.06	42.64	0.11	-	-	-	-	-	-	-	-	-	-	-	-	-	-	-	-	-	-
0	2483.03	5.88	0.08	-	-	-	-	-	-	-	-	-	-	-	-	-	-	-	-	-	-	-	-	-	-	-	-
4	2486.20	9.05	0.05	2.64	0.06	7.64	0.05	22.64	0.07	42.64	0.12	-	-	-	-	-	-	-	-	-	-	-	-	-	-	-	-
5	2489.41	12.27	0.05	2.64	0.07	7.64	0.06	22.64	0.00	27.64	0.05	42.64	0.15	-	-	-	-	-	-	-	-	-	-	-	-	-	-
6	2492.82	15.68	0.05	2.64	0.06	7.64	0.06	22.64	0.00	27.64	0.03	37.64	-0.04	42.64	0.14	-	-	-	-	-	-	-	-	-	-	-	-
7	2495.87	18.73	0.05	2.64	0.06	7.64	0.05	22.64	0.00	27.64	0.04	37.64	-0.04	42.64	0.28	47.64	0.13	-	-	-	-	-	-	-	-	-	-
8	2499.53	22.39	0.05	2.64	0.06	7.64	0.05	22.64	0.01	27.64	0.04	37.64	-0.04	42.64	0.24	47.64	0.04	52.64	0.20	-	-	-	-	-	-	-	-
9	2503.38	26.24	0.05	2.64	0.06	7.64	0.05	22.64	0.01	27.64	0.04	37.64	-0.04	42.64	0.28	47.64	0.04	52.64	0.19	62.64	0.86	-	-	-	-	-	-
10	2507.37	30.22	0.05	2.64	0.06	7.64	0.05	22.64	0.00	27.64	0.04	37.64	-0.04	42.64	0.28	47.64	0.04	52.64	0.18	57.64	0.19	62.64	0.89	-	-	-	-
11	2514.14	37.00	0.05	2.64	0.07	7.64	0.05	22.64	0.00	27.64	0.04	32.64	-0.04	37.64	0.06	42.64	0.03	47.64	0.05	52.64	0.28	57.64	0.19	62.64	1.04	-	-
12	2517.88	40.73	0.05	2.64	0.08	7.64	0.05	17.64	0.01	22.64	0.04	27.64	0.00	32.64	0.04	37.64	-0.01	42.64	0.10	47.64	0.23	52.64	0.19	57.64	0.19	62.64	1.19

Table S8. Results of the birth-death-shift models' comparison for the consensus tree of Pomacentridae. The models are sorted by $AICc$ in ascending order. We denote the $AICc$, $\Delta AICc$ and the model parameters (d: diversification rates, shift: shift date).

Nb	AICc	ΔAICc	d_0	$shift_1$	d_1	$shift_2$	d_2	$shift_3$	d_3	$shift_4$	d_4	$shift_5$	d_5	$shift_6$	d_6	$shift_7$	d_7	$shift_8$	d_8	$shift_9$	d_9	$shift_{10}$	d_{10}	$shift_{11}$	d_{11}
1	1582.45	0.00	0.06	6.49	0.08	-	-	-	-	-	-	-	-	-	-	-	-	-	-	-	-	-	-	-	-
0	1584.46	2.02	0.08	-	-	-	-	-	-	-	-	-	-	-	-	-	-	-	-	-	-	-	-	-	-
2	1584.75	2.30	0.06	6.49	0.06	26.49	0.09	-	-	-	-	-	-	-	-	-	-	-	-	-	-	-	-	-	-
3	1586.85	4.40	0.06	6.49	0.06	26.49	0.11	51.49	0.00	-	-	-	-	-	-	-	-	-	-	-	-	-	-	-	-
4	1590.14	7.69	0.06	6.49	0.07	21.49	0.05	26.49	0.10	51.49	0.00	-	-	-	-	-	-	-	-	-	-	-	-	-	-
5	1593.73	11.28	0.05	1.49	0.05	6.49	0.07	21.49	0.05	26.49	0.10	51.49	0.00	-	-	-	-	-	-	-	-	-	-	-	-
6	1597.09	14.65	0.05	1.49	0.06	6.49	0.06	16.49	0.00	21.49	0.10	26.49	0.11	51.49	0.00	-	-	-	-	-	-	-	-	-	-
7	1600.40	17.95	0.05	1.49	0.06	6.49	0.07	16.49	0.00	21.49	0.09	26.49	0.00	31.49	0.11	51.49	0.00	-	-	-	-	-	-	-	-
8	1603.99	21.55	0.05	1.49	0.06	6.49	0.07	16.49	0.00	21.49	0.09	26.49	0.00	31.49	0.08	46.49	0.21	51.49	0.00	-	-	-	-	-	-
9	1607.88	25.43	0.04	1.49	0.06	6.49	0.07	16.49	0.00	21.49	0.10	26.49	0.00	31.49	0.10	41.49	0.08	46.49	0.20	51.49	0.00	-	-	-	-
10	1611.26	28.82	0.02	1.49	0.05	6.49	0.09	16.49	0.00	21.49	0.10	26.49	0.00	31.49	0.22	36.49	0.00	41.49	0.06	46.49	0.20	51.49	0.00	-	-
11	1615.02	32.58	0.02	1.49	0.05	6.49	0.08	16.49	0.00	21.49	0.10	26.49	0.00	31.49	0.22	36.49	0.00	41.49	0.05	46.49	0.29	51.49	0.00	56.49	0.00

Table S9. Results of the birth-death-shift models' comparison for the consensus tree of Sparidae. The models are sorted by $AICc$ in ascending order. We denote the $AICc$, $\Delta AICc$ and the model parameters (d: diversification rates, shift: shift date).

Nb	AICc	ΔAICc	d_0	$shift_1$	d_1	$shift_2$	d_2	$shift_3$	d_3	$shift_4$	d_4	$shift_5$	d_5	$shift_6$	d_6	$shift_7$	d_7	$shift_8$	d_8	$shift_9$	d_9	$shift_{10}$	d_{10}	$shift_{11}$	d_{11}	$shift_{12}$	d_{12}
1	686.05	0.00	0.03	6.43	0.07	-	-	-	-	-	-	-	-	-	-	-	-	-	-	-	-	-	-	-	-	-	-
2	688.00	1.95	0.02	1.43	0.04	6.43	0.07	-	-	-	-	-	-	-	-	-	-	-	-	-	-	-	-	-	-	-	-
3	690.29	4.24	0.01	1.43	-0.05	6.43	0.09	36.43	0.00	-	-	-	-	-	-	-	-	-	-	-	-	-	-	-	-	-	-
0	690.49	4.45	0.06	-	-	-	-	-	-	-	-	-	-	-	-	-	-	-	-	-	-	-	-	-	-	-	-
4	693.77	7.72	0.01	1.43	-0.05	6.43	0.09	11.43	0.07	36.43	0.00	-	-	-	-	-	-	-	-	-	-	-	-	-	-	-	-
5	697.03	10.98	0.00	1.43	-0.05	6.43	0.08	11.43	0.06	31.43	0.03	36.43	-0.01	-	-	-	-	-	-	-	-	-	-	-	-	-	-
6	700.34	14.29	0.00	1.43	-0.05	6.43	0.09	11.43	0.04	16.43	0.03	31.43	0.06	36.43	0.00	-	-	-	-	-	-	-	-	-	-	-	-
7	703.66	17.62	0.00	1.43	-0.05	6.43	0.09	11.43	0.04	16.43	0.03	31.43	0.05	36.43	0.00	56.43	0.00	-	-	-	-	-	-	-	-	-	-
8	707.14	21.10	0.00	1.43	-0.05	6.43	0.09	11.43	0.04	16.43	0.03	31.43	0.05	36.43	0.00	51.43	0.00	56.43	0.00	-	-	-	-	-	-	-	-
9	710.81	24.77	0.00	1.43	-0.07	6.43	0.10	11.43	0.00	16.43	0.04	31.43	0.02	36.43	0.00	51.43	0.00	56.43	0.00	61.43	0.00	-	-	-	-	-	-
10	720.97	34.92	0.00	1.43	-0.07	6.43	0.09	11.43	0.00	16.43	0.04	21.43	0.01	31.43	0.00	36.43	0.00	51.43	-0.01	56.43	0.00	61.43	0.00	-	-	-	-
11	734.67	48.63	0.00	1.43	-0.07	6.43	0.09	11.43	0.00	16.43	0.04	21.43	0.01	31.43	0.00	36.43	0.00	46.43	-0.01	51.43	0.00	56.43	0.00	61.43	0.00	-	-
12	739.97	53.93	0.00	1.43	-0.06	6.43	0.09	11.43	0.00	16.43	0.05	21.43	0.02	31.43	0.00	36.43	0.00	41.43	-0.01	46.43	-0.01	51.43	-0.01	56.43	0.00	61.43	0.00

Table S10. Results of the environmental *birth-death* models' comparison for the consensus tree of Acanthuridae. For each variable, the models are sorted by $AICc$ in ascending order. We denote the $AICc$, $\Delta AICc$ and the model parameters (λ: speciation rate, μ: extinction rate, $\alpha_\lambda, \beta_\lambda$: parameters describing the relationship between the environmental variable and the speciation rate, α_μ, β_μ: parameters describing the relationship between the environmental variable and the extinction rate see Tab. 2 in main text).

Variable	Model	AICc	ΔAICc	λ	α_λ	β_λ	μ	α_μ	β_μ
Number of patches	Model 2.1	414.10	0.00	-	0.34	-0.01	0.16	-	-
	Model 2.2	417.40	3.29	-	0.18	-0.01	0.00	-	-
	Model 4.2	417.71	3.60	-	0.11	0.00	-	0.00	0.01
	Model 1	418.07	3.96	0.13	-	-	0.04	-	-
	Model 3.1	418.10	3.99	0.16	-	-	-	0.45	0.00
	Model 4.1	420.56	6.46	-	-0.02	0.00	-	-0.12	0.00
	Model 3.2	421.00	6.90	0.10	-	-	-	0.00	-0.05
Total area	Model 2.2	416.83	0.00	-	0.15	0.00	0.00	-	-
	Model 1	418.07	1.24	0.13	-	-	0.04	-	-
	Model 4.1	422.21	5.37	-	0.05	0.00	-	0.01	0.00
	Model 2.1	423.10	6.27	-	0.05	0.00	0.01	-	-
	Model 4.2	430.00	13.17	-	0.05	0.00	-	0.01	0.00
	Model 3.2	660.32	243.49	0.01	-	-	-	0.00	0.00
	Model 3.1	966.97	550.14	0.00	-	-	-	0.00	0.00
Temperature	Model 3.2	415.43	0.00	0.12	-	-	-	0.01	0.07
	Model 2.2	416.98	1.55	-	0.14	-0.03	0.00	-	-
	Model 1	418.07	2.64	0.13	-	-	0.04	-	-
	Model 3.1	418.17	2.75	0.12	-	-	-	-0.01	0.01
	Model 4.2	419.26	3.83	-	0.13	-0.03	-	0.00	-0.04
	Model 4.1	419.36	3.93	-	0.07	0.02	-	-0.02	0.03
	Model 2.1	420.56	5.13	-	0.11	0.00	0.00	-	-

Table S11. Results of the environmental *birth-death* models comparison for the consensus tree of Balistoidae. For each variable, the models are sorted by $AICc$ in ascending order. We denote the $AICc$, $\Delta AICc$ and the model parameters (λ: speciation rate, μ: extinction rate, $\alpha_\lambda, \beta_\lambda$: parameters describing the relationship between the environmental variable and the speciation rate, α_μ, β_μ: parameters describing the relationship between the environmental variable and the extinction rate see Tab. 2 in main text).

Variable	Model	AICc	ΔAICc	λ	α_λ	β_λ	μ	α_μ	β_μ
Number of patches	Model 3.1	574.62	0.00	0.25	-	-	-	0.93	-0.01
	Model 1	578.47	3.86	0.17	-	-	0.09	-	-
	Model 2.1	578.60	3.98	-	0.11	0.00	0.20	-	-
	Model 4.1	580.35	5.73	-	-0.28	0.01	-	0.37	0.00
	Model 2.2	580.40	5.78	-	0.17	0.00	0.06	-	-
	Model 4.2	583.42	8.81	-	0.20	-0.01	-	0.08	-0.14
	Model 3.2	584.20	9.58	0.11	-	-	-	0.01	-0.04
Total area	Model 1	578.47	0.00	0.17	-	-	0.09	-	-
	Model 4.1	583.03	4.55	-	0.05	0.00	-	0.01	0.00
	Model 3.1	583.82	5.35	0.15	-	-	-	-0.12	0.00
	Model 2.1	587.66	9.19	-	0.05	0.00	0.01	-	-
	Model 4.2	591.87	13.40	-	0.05	0.00	-	0.01	0.00
	Model 2.2	609.97	31.49	-	0.06	0.00	0.00	-	-
	Model 3.2	950.54	372.07	0.01	-	-	-	0.00	0.00
Temperature	Model 1	578.47	0.00	0.17	-	-	0.09	-	-
	Model 3.2	578.70	0.22	0.12	-	-	-	0.00	0.22
	Model 4.2	579.54	1.07	-	0.27	-0.04	-	0.53	-0.20
	Model 2.2	580.42	1.95	-	0.16	-0.01	0.06	-	-
	Model 2.1	580.57	2.09	-	0.17	0.00	0.11	-	-
	Model 3.1	581.65	3.17	0.13	-	-	-	-0.01	0.01
	Model 4.1	582.77	4.29	-	0.17	0.00	-	0.11	0.00

Table S12. Results of the environmental *birth-death* models comparison for the consensus tree of Carangoidae. For each variable, the models are sorted by $AICc$ in ascending order. We denote the $AICc$, $\Delta AICc$ and the model parameters (λ: speciation rate, μ: extinction rate, $\alpha_\lambda, \beta_\lambda$: parameters describing the relationship between the environmental variable and the speciation rate, α_μ, β_μ: parameters describing the relationship between the environmental variable and the extinction rate see Tab. 2 in main text).

Variable	Model	AICc	ΔAICc	λ	α_λ	β_λ	μ	α_μ	β_μ
Number of patches	Model 1	1021.63	0.00	0.06	-	-	0.00	-	-
	Model 2.2	1022.55	0.92	-	0.07	0.00	0.00	-	-
	Model 3.2	1022.89	1.26	0.06	-	-	-	0.00	0.01
	Model 3.1	1022.96	1.33	0.06	-	-	-	0.05	0.00
	Model 2.1	1023.76	2.13	-	0.06	0.00	0.01	-	-
	Model 4.2	1024.68	3.05	-	0.07	0.00	-	0.01	-0.39
	Model 4.1	1025.41	3.78	-	0.05	0.00	-	0.00	0.00
Total area	Model 1	1021.63	0.00	0.06	-	-	0.00	-	-
	Model 3.1	1023.48	1.85	0.06	-	-	-	-0.01	0.00
	Model 2.1	1023.86	2.23	-	0.05	0.00	0.01	-	-
	Model 4.1	1026.03	4.40	-	0.05	0.00	-	0.01	0.00
	Model 2.2	1026.81	5.18	-	0.05	0.00	0.01	-	-
	Model 4.2	1027.88	6.25	-	0.05	0.00	-	0.01	0.00
	Model 3.2	1378.07	356.44	0.01	-	-	-	0.00	0.00
Temperature	Model 1	1021.63	0.00	0.06	-	-	0.00	-	-
	Model 2.2	1022.67	1.04	-	0.06	-0.01	0.00	-	-
	Model 3.2	1022.89	1.26	0.06	-	-	-	0.00	0.04
	Model 3.1	1023.27	1.64	0.06	-	-	-	0.00	0.00
	Model 2.1	1023.29	1.66	-	0.06	0.00	0.00	-	-
	Model 4.2	1024.80	3.17	-	0.06	-0.01	-	0.00	0.06
	Model 4.1	1028.07	6.44	-	0.06	0.00	-	0.02	0.00

Table S13. Results of the environmental *birth-death* models' comparison for the consensus tree of Chaetodontidae. For each variable, the models are sorted by $AICc$ in ascending order. We denote the $AICc$, $\Delta AICc$ and the model parameters (λ: speciation rate, μ: extinction rate, $\alpha_\lambda, \beta_\lambda$: parameters describing the relationship between the environmental variable and the speciation rate, α_μ, β_μ: parameters describing the relationship between the environmental variable and the extinction rate see Tab. 2 in main text).

Variable	Model	AICc	ΔAICc	λ	α_λ	β_λ	μ	α_μ	β_μ
Number of patches	Model 1	548.31	0.00	0.26	-	-	0.13	-	-
	Model 2.2	549.37	1.05	-	0.33	0.00	0.09	-	-
	Model 3.1	550.10	1.79	0.25	-	-	-	-0.02	0.00
	Model 2.1	550.12	1.81	-	0.08	0.00	0.21	-	-
	Model 4.2	551.31	3.00	-	0.47	-0.01	-	0.46	-0.13
	Model 4.1	551.72	3.41	-	0.06	0.00	-	0.01	0.00
	Model 3.2	554.22	5.91	0.19	-	-	-	0.02	-0.08
Total area	Model 1	548.31	0.00	0.26	-	-	0.13	-	-
	Model 3.1	549.23	0.92	0.23	-	-	-	-0.17	0.00
	Model 4.1	552.27	3.95	-	0.05	0.00	-	0.01	0.00
	Model 2.1	561.35	13.03	-	0.05	0.00	0.00	-	-
	Model 4.2	570.88	22.57	-	0.05	0.00	-	0.01	0.00
	Model 2.2	602.75	54.44	-	0.06	0.00	0.00	-	-
	Model 3.2	1051.15	502.83	0.01	-	-	-	0.00	0.00
Temperature	Model 3.2	548.31	0.00	0.23	-	-	-	0.04	0.06
	Model 1	548.31	0.01	0.26	-	-	0.13	-	-
	Model 3.1	548.82	0.51	0.23	-	-	-	-0.02	0.02
	Model 2.2	549.10	0.79	-	0.25	-0.03	0.03	-	-
	Model 4.1	549.68	1.38	-	0.03	0.14	-	0.12	0.12
	Model 2.1	550.36	2.05	-	0.27	-0.01	0.09	-	-
	Model 4.2	551.33	3.03	-	0.25	-0.05	-	0.00	-0.02

Table S14. Results of the environmental *birth-death* models' comparison for the consensus tree of Haemulinae. For each variable, the models are sorted by $AICc$ in ascending order. We denote the $AICc$, $\Delta AICc$ and the model parameters (λ: speciation rate, μ: extinction rate, $\alpha_\lambda, \beta_\lambda$: parameters describing the relationship between the environmental variable and the speciation rate, α_μ, β_μ: parameters describing the relationship between the environmental variable and the extinction rate see Tab. 2 in main text).

Variable	Model	AICc	ΔAICc	λ	α_λ	β_λ	μ	α_μ	β_μ
Number of patches	Model 1	380.76	0.00	0.08	-	-	0.00	-	-
	Model 2.1	382.55	1.80	-	0.02	0.00	0.00	-	-
	Model 2.2	382.75	1.99	-	0.07	0.00	0.00	-	-
	Model 3.2	383.02	2.27	0.08	-	-	-	0.00	-0.02
	Model 3.1	383.02	2.27	0.08	-	-	-	0.00	0.00
	Model 4.2	383.54	2.78	-	0.02	0.02	-	0.02	0.02
	Model 4.1	385.09	4.33	-	0.05	0.00	-	0.01	0.00
Total area	Model 1	380.76	0.00	0.08	-	-	0.00	-	-
	Model 2.1	380.85	0.09	-	0.05	0.00	0.01	-	-
	Model 2.2	382.70	1.94	-	0.07	0.00	0.00	-	-
	Model 4.1	384.16	3.41	-	0.05	0.00	-	0.01	0.00
	Model 4.2	385.20	4.44	-	0.05	0.00	-	0.01	0.00
	Model 3.1	386.79	6.03	0.10	-	-	-	-0.07	0.00
	Model 3.2	579.87	199.11	0.01	-	-	-	0.00	0.00
Temperature	Model 4.1	378.32	0.00	-	-0.06	0.05	-	0.06	0.02
	Model 2.1	378.44	0.12	-	0.01	0.02	0.11	-	-
	Model 1	380.76	2.44	0.08	-	-	0.00	-	-
	Model 2.2	382.56	4.24	-	0.08	0.01	0.02	-	-
	Model 3.2	383.02	4.70	0.08	-	-	-	0.00	-0.03
	Model 3.1	383.02	4.71	0.08	-	-	-	0.00	0.00
	Model 4.2	384.80	6.49	-	0.07	0.01	-	0.01	0.02

Table S15. Results of the environmental *birth-death* models' comparison for the consensus tree of Holocentridae. For each variable, the models are sorted by $AICc$ in ascending order. We denote the $AICc$, $\Delta AICc$ and the model parameters (λ: speciation rate, μ: extinction rate, $\alpha_\lambda, \beta_\lambda$: parameters describing the relationship between the environmental variable and the speciation rate, α_μ, β_μ: parameters describing the relationship between the environmental variable and the extinction rate see Tab. 2 in main text).

Variable	Model	AICc	ΔAICc	λ	α_λ	β_λ	μ	α_μ	β_μ
Number of patches	Model 2.1	307.67	0.00	-	0.37	-0.01	0.17	-	-
	Model 2.2	309.30	1.62	-	0.31	-0.01	0.01	-	-
	Model 4.1	309.78	2.10	-	-0.43	0.01	-	0.05	0.00
	Model 4.2	311.37	3.70	-	0.28	-0.01	-	0.01	0.00
	Model 3.1	312.82	5.14	0.19	-	-	-	0.73	-0.01
	Model 1	313.67	6.00	0.18	-	-	0.13	-	-
	Model 3.2	322.13	14.46	0.10	-	-	-	0.00	-0.06
Total area	Model 1	313.67	0.00	0.18	-	-	0.13	-	-
	Model 4.1	315.28	1.61	-	0.05	0.00	-	0.01	0.00
	Model 2.1	326.02	12.34	-	0.05	0.00	0.01	-	-
	Model 4.2	336.22	22.55	-	0.05	0.00	-	0.01	0.00
	Model 2.2	336.43	22.76	-	0.06	0.00	0.00	-	-
	Model 3.2	496.57	182.89	0.01	-	-	-	0.00	0.00
	Model 3.1	715.35	401.68	0.00	-	-	-	0.01	0.00
Temperature	Model 4.1	302.78	0.00	-	-0.07	0.08	-	-0.07	0.08
	Model 2.2	310.04	7.26	-	0.17	-0.04	0.01	-	-
	Model 3.1	310.45	7.67	0.15	-	-	-	-0.01	0.01
	Model 3.2	310.82	8.04	0.15	-	-	-	0.05	0.02
	Model 4.2	312.10	9.32	-	0.16	-0.03	-	0.01	0.02
	Model 1	313.67	10.89	0.18	-	-	0.13	-	-
	Model 2.1	314.23	11.45	-	0.18	-0.01	0.01	-	-

Table S16. Results of the environmental *birth-death* models' comparison for the consensus tree of Labridae. For each variable, the models are sorted by $AICc$ in ascending order. We denote the $AICc$, $\Delta AICc$ and the model parameters (λ: speciation rate, μ: extinction rate, $\alpha_\lambda, \beta_\lambda$: parameters describing the relationship between the environmental variable and the speciation rate, α_μ, β_μ: parameters describing the relationship between the environmental variable and the extinction rate see Tab. 2 in main text).

Variable	Model	AICc	ΔAICc	λ	α_λ	β_λ	μ	α_μ	β_μ
Number of patches	Model 1	2479.77	0.00	0.11	-	-	0.02	-	-
	Model 2.1	2480.82	1.05	-	0.05	0.00	0.01	-	-
	Model 4.1	2481.01	1.24	-	0.05	0.00	-	0.00	0.00
	Model 2.2	2481.19	1.42	-	0.12	0.00	0.00	-	-
	Model 3.1	2481.26	1.49	0.11	-	-	-	-0.04	0.00
	Model 4.2	2483.24	3.47	-	0.12	0.00	-	0.03	-0.14
	Model 3.2	2483.47	3.70	0.10	-	-	-	0.00	-0.02
Total area	Model 1	2479.77	0.00	0.11	-	-	0.02	-	-
	Model 2.2	2481.34	1.57	-	0.12	0.00	0.00	-	-
	Model 3.1	2481.84	2.07	0.12	-	-	-	-0.07	0.00
	Model 4.1	2482.26	2.49	-	0.05	0.00	-	0.01	0.00
	Model 2.1	2489.89	10.12	-	0.05	0.00	0.01	-	-
	Model 4.2	2491.46	11.69	-	0.05	0.00	-	0.01	0.00
	Model 3.2	4170.94	1691.17	0.00	-	-	-	0.00	0.00
Temperature	Model 1	2479.77	0.00	0.11	-	-	0.02	-	-
	Model 3.1	2480.19	0.42	0.11	-	-	-	0.00	0.00
	Model 2.2	2480.51	0.74	-	0.11	-0.01	0.00	-	-
	Model 4.1	2480.69	0.92	-	0.07	0.01	-	-0.01	0.01
	Model 3.2	2481.08	1.31	0.11	-	-	-	0.01	0.02
	Model 2.1	2481.29	1.52	-	0.12	0.00	0.00	-	-
	Model 4.2	2482.56	2.79	-	0.11	-0.01	-	0.00	-0.01

Table S17. Results of the environmental *birth-death* models' comparison for the consensus tree of Pomacentridae. For each variable, the models are sorted by $AICc$ in ascending order. We denote the $AICc$, $\Delta AICc$ and the model parameters (λ: speciation rate, μ: extinction rate, $\alpha_\lambda, \beta_\lambda$: parameters describing the relationship between the environmental variable and the speciation rate, α_μ, β_μ: parameters describing the relationship between the environmental variable and the extinction rate see Tab. 2 in main text).

Variable	Model	AICc	ΔAICc	λ	α_λ	β_λ	μ	α_μ	β_μ
Number of patches	Model 3.1	1505.69	0.00	0.14	-	-	-	-0.14	0.00
	Model 1	1506.44	0.74	0.15	-	-	0.04	-	-
	Model 4.1	1506.67	0.98	-	-0.12	0.00	-	-0.13	0.00
	Model 2.1	1507.74	2.05	-	-0.06	0.00	0.06	-	-
	Model 2.2	1508.25	2.55	-	0.17	0.00	0.01	-	-
	Model 4.2	1508.46	2.77	-	0.07	0.01	-	0.03	0.01
	Model 3.2	1511.25	5.56	0.12	-	-	-	0.00	-0.06
Total area	Model 1	1506.44	0.00	0.15	-	-	0.04	-	-
	Model 2.2	1507.99	1.55	-	0.16	0.00	0.02	-	-
	Model 4.1	1509.02	2.58	-	0.05	0.00	-	0.01	0.00
	Model 2.1	1519.08	12.65	-	0.05	0.00	0.01	-	-
	Model 4.2	1523.38	16.94	-	0.05	0.00	-	0.00	0.00
	Model 3.1	2257.61	751.17	0.03	-	-	-	-0.01	0.00
	Model 3.2	2669.83	1163.39	0.00	-	-	-	0.00	0.00
Temperature	Model 3.1	1506.11	0.00	0.14	-	-	-	-0.01	0.01
	Model 1	1506.44	0.33	0.15	-	-	0.04	-	-
	Model 2.1	1506.75	0.64	-	0.16	-0.01	0.00	-	-
	Model 2.2	1507.12	1.00	-	0.14	-0.02	0.00	-	-
	Model 3.2	1507.80	1.68	0.14	-	-	-	0.03	0.02
	Model 4.1	1507.94	1.83	-	0.11	0.01	-	-0.01	0.02
	Model 4.2	1509.19	3.08	-	0.14	-0.02	-	0.00	0.00

Table S18. Results of the environmental *birth-death* models' comparison for the consensus tree of Sparidae. For each variable, the models are sorted by $AICc$ in ascending order. We denote the $AICc$, $\Delta AICc$ and the model parameters (λ: speciation rate, μ: extinction rate, $\alpha_\lambda, \beta_\lambda$: parameters describing the relationship between the environmental variable and the speciation rate, α_μ, β_μ: parameters describing the relationship between the environmental variable and the extinction rate see Tab. 2 in main text).

Variable	Model	AICc	ΔAICc	λ	α_λ	β_λ	μ	α_μ	β_μ
Number of patches	Model 2.1	685.28	0.00	-	0.06	0.00	0.00	-	-
	Model 4.2	687.11	1.83	-	0.03	0.01	-	0.00	0.02
	Model 1	687.18	1.90	0.07	-	-	0.00	-	-
	Model 2.2	688.69	3.42	-	0.06	0.00	0.00	-	-
	Model 3.2	689.32	4.04	0.07	-	-	-	0.00	-0.02
	Model 3.1	689.62	4.34	0.07	-	-	-	0.02	0.00
	Model 4.1	690.54	5.27	-	0.05	0.00	-	0.01	0.00
Total area	Model 2.1	684.42	0.00	-	0.05	0.00	0.01	-	-
	Model 1	687.18	2.76	0.07	-	-	0.00	-	-
	Model 2.2	688.50	4.09	-	0.06	0.00	0.00	-	-
	Model 4.2	689.37	4.96	-	0.05	0.00	-	0.01	0.00
	Model 4.1	689.98	5.56	-	0.05	0.00	-	0.01	0.00
	Model 3.2	969.79	285.37	0.01	-	-	-	0.00	0.00
	Model 3.1	1418.50	734.08	0.00	-	-	-	0.00	0.00
Temperature	Model 2.1	686.17	0.00	-	0.03	0.01	0.00	-	-
	Model 1	687.18	1.01	0.07	-	-	0.00	-	-
	Model 4.2	687.70	1.53	-	0.05	0.03	-	0.00	0.08
	Model 4.1	687.90	1.73	-	0.03	0.01	-	0.00	0.00
	Model 2.2	689.07	2.91	-	0.07	0.00	0.00	-	-
	Model 3.2	689.32	3.15	0.07	-	-	-	0.00	-0.03
	Model 3.1	689.90	3.73	0.07	-	-	-	0.00	0.00

Figure S1. Results of the model's comparison on each taxon's posterior distribution of trees. Each histogram represents the frequency at which each model has been selected by the AICc approach. The green bars represent the best models and the grey bars represent the models that have a $\Delta AICc$ below 2. The first column represents the Number of patch dependant models. The second column represents the total reef area dependant models. The third column represents the temperature dependant models. The Fourth column represents *birth-death-shift* models. **Aca:** Acanthuridae; **Bal:** Balistoidae; **Car:** Carangoidae; **Cha:** Chaetodontidae; **Hae:** Haemulinae, **Hol:** Holocentridae; **Lab:** labrinae; **Pom:** Pomacentridae; **Spa:** Sparidae.

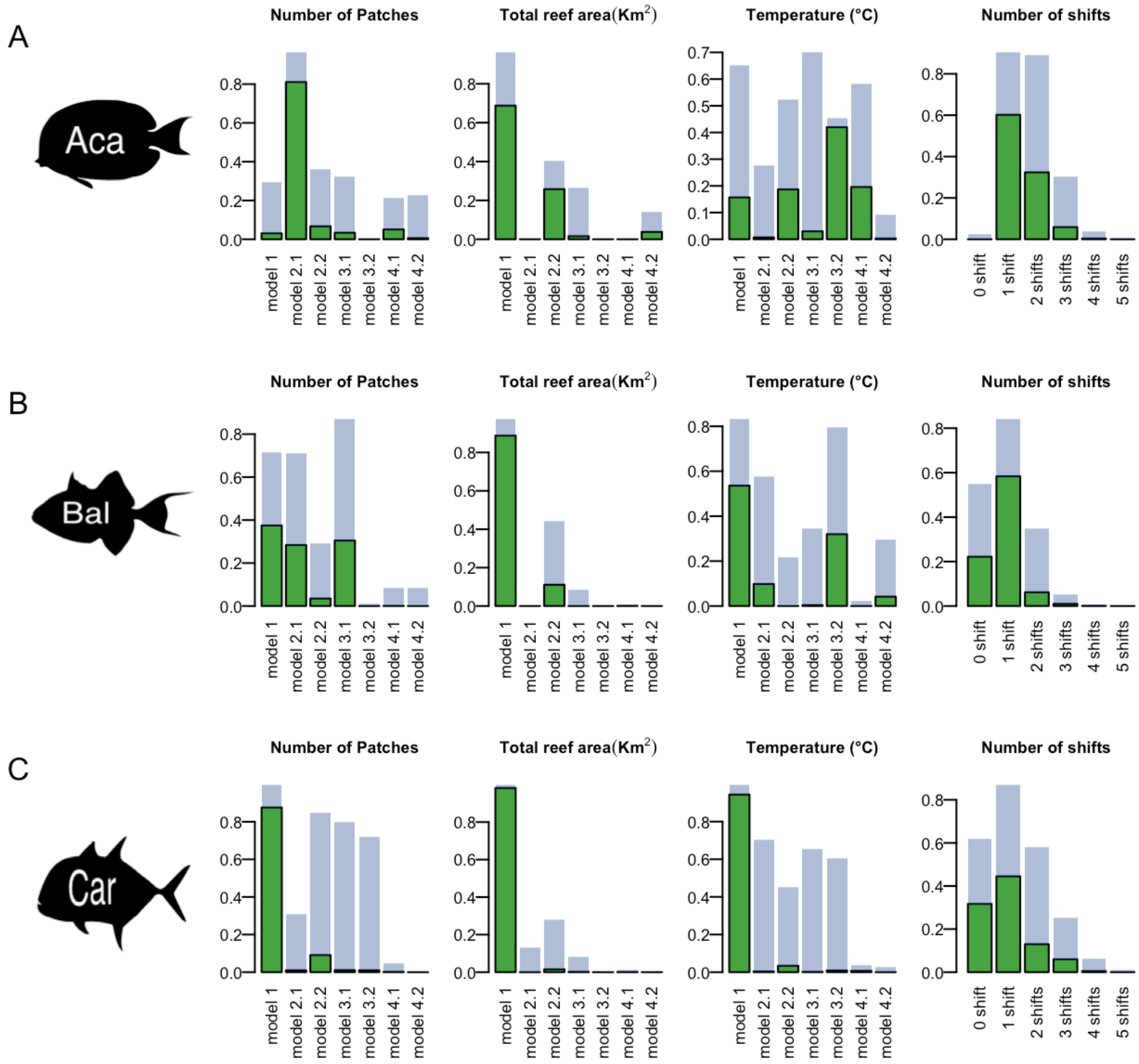

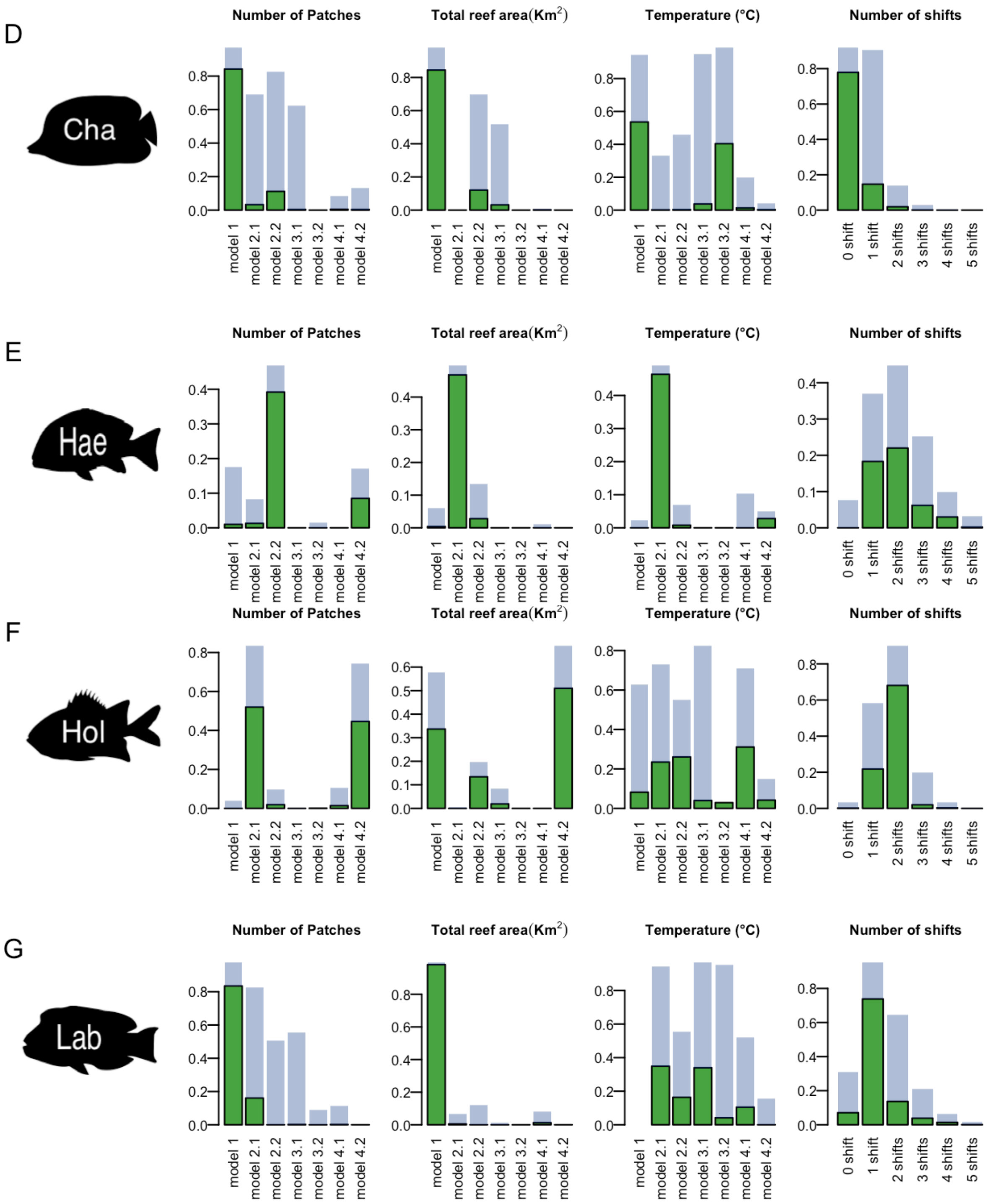
D
Cha
Number of Patches
Total reef area(Km^2)
Temperature (°C)
Number of shifts
E
Hae
Number of Patches
Total reef area(Km^2)
Temperature (°C)
Number of shifts
F
Hol
Number of Patches
Total reef area(Km^2)
Temperature (°C)
Number of shifts
G
Lab
Number of Patches
Total reef area(Km^2)
Temperature (°C)
Number of shifts
model 1
model 2.1
model 2.2
model 3.1
model 3.2
model 4.1
model 4.2
0 shift
1 shift
2 shifts
3 shifts
4 shifts
5 shifts

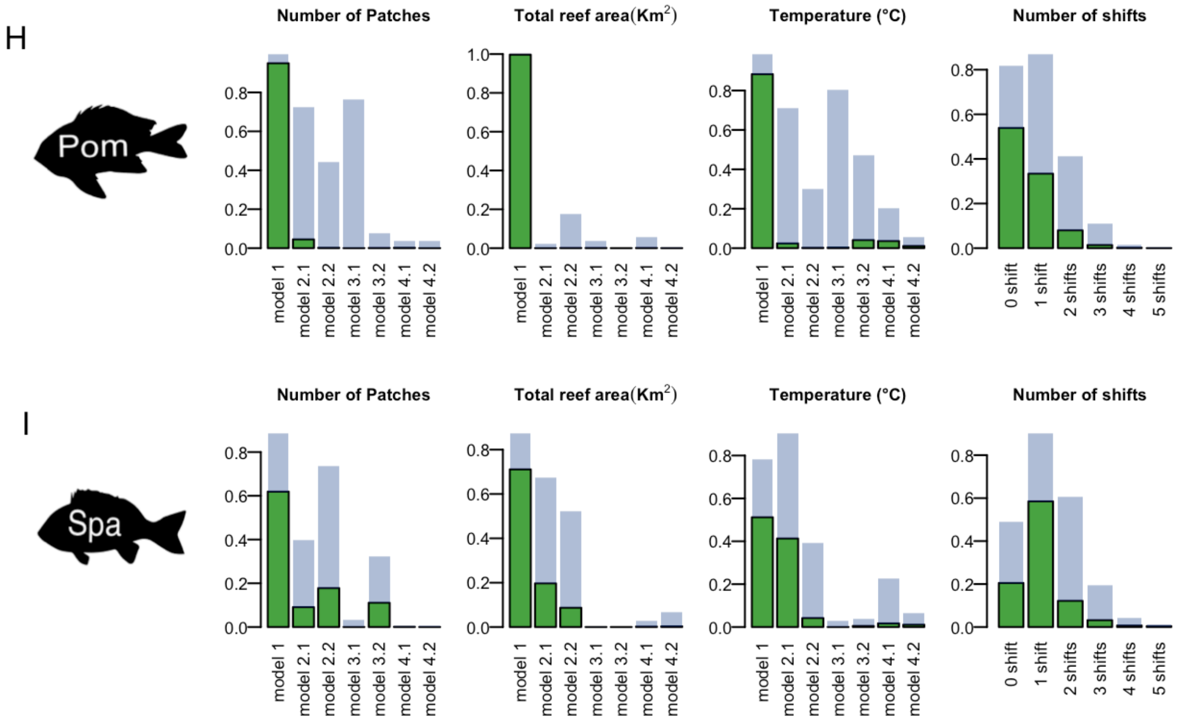
H
Pom
Number of Patches
Total reef area(Km2)
Temperature (°C)
Number of shifts
model 1
model 2.1
model 2.2
model 3.1
model 3.2
model 4.1
model 4.2
0 shift
1 shift
2 shifts
3 shifts
4 shifts
5 shifts
I
Spa
Number of Patches
Total reef area(Km2)
Temperature (°C)
Number of shifts
